# *Zbtb20* modulates the sequential generation of neuronal layers in developing cortex

**DOI:** 10.1186/s13041-016-0242-2

**Published:** 2016-06-09

**Authors:** Anton B. Tonchev, Tran Cong Tuoc, Eva H. Rosenthal, Michèle Studer, Anastassia Stoykova

**Affiliations:** Molecular Developmental Neurobiology Laboratory, Max Planck Institute of Biophysical Chemistry, Am Fassberg, 37077 Gottingen, Germany; Center for Nanoscale Microscopy and Molecular Physiology of the Brain (CNMPB), 37075 Göttingen, Germany; Department of Anatomy, Histology and Embryology, Medical University-Varna, Varna, Bulgaria; Molecular Neurobiology Group, Institute of Neuroanatomy, University of Goettingen Medical Center, Goettingen, Germany; University Nice Sophia Antipolis, iBV, UMR 7277, F-06108 Nice, France; Inserm, iBV, U1091, F-06108 Nice, France

**Keywords:** Zbtb20, Development, Neocortex, Temporal identity, Transcription factor

## Abstract

**Background:**

During corticogenesis, genetic programs encoded in progenitor cells at different developmental stages and inherited in postmitotic neurons specify distinct layer and area identities. Transcription factor Zbtb20 has been shown to play a role for hippocampal development but whether it is implicated in mammalian neocortical morphogenesis remains unknown.

**Results:**

Here, we report that during embyogenesis transcription factor *Zbtb20* has a dynamic spatio-temporal expression pattern in mitotic cortical progenitors through which it modulates the sequential generation of cortical neuronal layer identities. *Zbtb20* knock out mice exhibited enhanced populations of early born L6-L4 neuronal subtypes and a dramatic reduction of the late born L3/L2 neurons. This defect was due to a temporal misbalance in the production of earlier versus later born neurons, leading to a progressive diminishing of the progenitor pool for the generation of L3-L2 neurons. Zbtb20 implements these temporal effects in part by binding to promoter of the orphan nuclear receptor *CoupTF1/Nr2f1.* In addition to its effects exerted in cortical progenitors, the postmitotic expression of Zbtb20 in L3/L2 neurons starting at birth may contribute to their proper differentiation and migration.

**Conclusions:**

Our findings reveal *Zbtb20* as a novel temporal regulator for the generation of layer-specific neuronal identities.

**Electronic supplementary material:**

The online version of this article (doi:10.1186/s13041-016-0242-2) contains supplementary material, which is available to authorized users.

## Background

The mammalian neocortex (Ncx), in which neurons are arranged radially in six layers and tangentially in numerous functional domains, is a recent acquisition in brain evolution. During development, the majority of cortical glutamatergic neurons are generated by radial glial cells (RGCs) in the germinative ventricular (VZ) and subventricular (SVZ) zone of the dorsolateral pallium. Generation of neuronal sets with a layer-specific identity depends on an intrinsically encoded genetic program and environmental cues acting during the S-phase of the mitotic cycle [1]. Neurons with different fates are produced according to an “inside-first outside-last” schedule: first, lower layer (LL) neurons (L6/L5), followed by generation of the upper layer (UL) neurons (L4/L3/L2). During mouse development, the layer specific neuronal subtypes are generated throughout embryonic (E) stages E10.5 – E17.5 in partially overlapping time windows with a peak for generation at E11.5 for L1, E12.5 (L6), E13.5 (L5), E14.5 (L4) and E16.5-E17.5 (L3-L2) [2–5]. The birthdate of cortical neurons is directly also related to their projection identity. Thus, while the early born L6 and L5 neurons extend outside the brain thalamocortical (TCA) and corticospinal motor neuron (CSMN) projections, the late-born UL neurons make interhemispheric (callosal) projections inside the brain [6]. Increasing recent evidence support the view that the precise temporal programs for production of LL and UL neuronal fates relies on intrinsic mechanisms in early and late progenitors, respectively, characterized by specific combinatorial expression of TFs at distinct developmental time points [7–13]. For instance, suppression of the expression of *Foxg1* at E10.5 is required to make a switch from generation of reelin-positive Cajal-Retzius cells, located in the marginal zone (MZ) of the cortex, to the production of neuronal subsets located in cortical plate (CP) [14]. Furthermore, while *Fezf2* and *Otx1* expression in apical VZ progenitors controls the fate specification of the LL neurons [15, 16], the expression of *Svet1, Cux1* and *Cux2* during later stages of neurogenesis in SVZ progenitors seems to specify UL neuronal fate [17–20]. The expression of TFs in postmitotic CP neurons may regulate through feedback signalling mechanism the progenitor progeny in the germinative zone [21] or the fate of the postmitotic neurons [7–13].

Neurons with distinct morphology, connectivity, neurotransmitter usage and function are tangentially organized in numerous functional domains, implicating that mechanisms of layer and area formation are interrelated. According to the current view, cortical arealization is presaged by encoded positional information (“protomap”) through graded expression of sets of TFs along the anteroposterior and mediolateral axis in the two germinative zones of the neocortex [5, 22]. Disruption of the graded expression of such TFs in VZ/SVZ leads to severe defects in the areal size and location in the Ncx [23–25]. As recently shown, affecting the intrinsic genetic mechanisms encoded by the graded expression of TF Pax6 in cortical progenitors results in an altered size of cortical somatosensory (SS) area and in parallel alterations in the sensory thalamus involving selective death of neurons in particular thalamic nuclei. Consequently, a new type of “top-down plasticity” driven by competition-mediated axon elimination and neuronal apoptosis re-patterns the sensory thalamus [26].

In a microarray screen aimed to find out genes with graded expression in the developing cortex, we identified TF *Zbtb20* as a gene showing caudal-high to rostral-low expression gradient in VZ of E16.5 cortex, and subsequently maintains a restricted high expression in the adult hippocampus (Hi) [27]. The gene *Zbtb20* (also named DPZF [28], HOF [29] or ZNF288) encodes a TF, belonging to the POK-family of BTB zinc finger transcriptional repressors, implicated in developmental processes and cancer [30]. Zbtb20 has been localized exclusively in immature post-mitotic neurons in Hi and migrating granule cell precursors of DG [29]. Previous research, using both gain-of-function (GOF) [31–33] and loss-of-function (LOF) [34, 35] approaches, has described the important role of TF *Zbtb20* in specifying the medial pallium, the anlage of the Hi formation.

Recent studies have implicated *Zbtb20* mutations in human neurodevelopmental syndromes associated with behavioral abnormalities, including intellectual disability [36], Primrose syndrome [37], autism [38], and schizophrenia [39]. The reported alterations in brain morphology in these disorders suggest possible cortical involvement beyond the hippocampus. However, no data are presently available on the involvement of Zbtb20 in neocortical morphogenesis.

In this study, we present first evidence that TF *Zbtb20* exerts a dynamic expression in the germinative zones of the cortex (pallium), marks specifically the uppermost L3-L2 cortical neurons and exerts a crucial control in the timely generation of distinct neuronal fates throughout cortical neurogenesis.

## Results

### Dynamic Zbtb20 expression in telencephalic progenitors

**Fig. 1:**
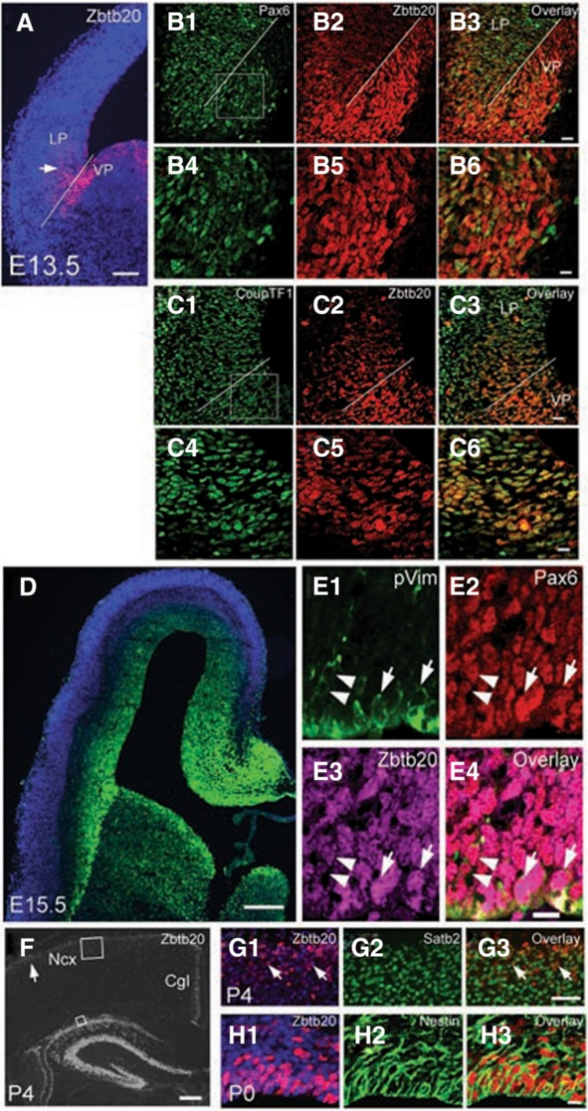
Expression of TF Zbtb20 in the developing pallium. a Immunostaining for Zbtb20 at E13.5 demarcates a strong signal just ventral to the cortico-striatal sulcus (white line) in VP and a weaker gradient in LP (*arrow*) gradually disappearing toward DP. **b1-b6** Double-labelling for Pax6 (b1/b4) and Zbtb20 (b2/b5), and an overlay (b3/b6) in VZ of LP and VP at E13.5. **c1-c6** Double-labelling for CoupTF1 (c1/c4) and Zbtb20 (c2/c5), and an overlay (c3/c6) in VZ of LP and VP at E13.5. The cortico-striatal sulcus is depicted by a white line. **d** Zbtb20 IHC on a cross brain section at stage E15.5 demonstrates strong immunosignal in germinative zones of both pallium and subpallim. **e1-e4** Triple IHC with antibodies for phospho-vimentin (pVim, E1), Pax6 (e2) and Zbtb20 (e3), and an overlay (e4) in E16.5 DP. Arrows depict triple-positive cells at the apical surface, arrowheads depict a more basally located dividing cell. **f** IHC for Zbtb20 on coronal WT brain sections at P4. The arrow points to the Zbtb20 expression in the uppermost neocortical layers. The frames depict the position of the micrographs shown in G1-G3. **g1-g3** Double immunostaining for Zbtb20 (g1, counterstained by DAPI) and Satb2 (g2), and an overlay (g3) on P4 brain cross sections. Arrows depict double-positive cells in the uppermost layers. **h1-h3** Double immunostaining for Zbtb20 (H1, counterstained by DAPI) and Nestin (h2), and an overlay (h3) on P0 brain cross sections depicts almost complete co-staining in VZ/SVZ. Cgl, cingulate cortex; DP, dorsal pallium; LP, lateral pallium; Ncx, neocortex; VP, ventral pallium. Scale bars: **a**, 100 μm; **b3/c3**, 20 μm; **d**, 200 μm; **e4**, 10 μm; **f**, 200 μm; **g3**, 50 μm; **b6/c6/h3**, 10 μm

By E11.5 a gradient of *Zbtb20*-lacZ activity was evident in the VZ of the lateral pallium (LP) of the heterozygous *Zbtb20*^*lacZ/+*^ embryos (Additional file 1: Figure S1A, arrow). Between E12.5 and E13.5, Zbtb20 immunostaining confirmed a strong signal in both the ventral (VP) and lateral (LP) pallium (Fig. 1a, arrow). Double staining for Zbtb20 and the pallial progenitor marker Pax6 revealed a nearly complete co-expression at E13.5 (Fig. 1b1-b6) in LP (101 out of 101 assayed Zbtb20^+^ cells co-expressed Pax6 (100 %, *n* = 3). In VP, however, only 40 % of the Zbtb20^+^ cells co-expressed Pax6 (59 out of 148 Zbtb20^+^ cells, *n* = 3). Notably, co-labelling for Zbtb20 and TF CoupTF1, which at this stage is expressed by both pallial and subpallial progenitors, showed a complete co-expression in LP and VP (143 out of 143 Zbtb20+ cells in LP/VP (*n* = 3) co-expressed CoupTF1 (Fig. 1c1-c6).

By E14.5-E15.5 the expression of *Zbtb20* was spread throughout the entire pallial VZ (Fig. 1d; Additional file 1: Figure S1B, arrows). Co-staining for Zbtb20, RGC marker Pax6 and the mitotic marker phosphorylated vimentin (pVim) confirmed that TF Zbtb20 is expressed in dividing RGCs at the apical surface of VZ (Fig. 1e1-e4, arrows; 90 out of 93 assayed pVim^+^ cells on the apical surface were co-labeled for Zbtb20, 97 %, *n* = 3).

At early postnatal (P) stages (P4), Zbtb20 immunosignal was evident in the most superficial layers of LP/DP (Fig. 1f, arrow), where Zbtb20^+^ cells co-expressed Satb2 (Fig. 1g1-g3, arrows; 102 out of 129 assayed Zbtb20^+^ cells were co-labeled for Satb2, 79 %, *n* = 3) and Brn2 (Additional file 2: Figure S2A1-A3, arrows; 111 out of 121 assayed Zbtb20^+^ cells were co-labeled for Brn2, 93 %, n = 3), markers of UL neocortical neurons [40, 41]. However, Zbtb20 did not co-localize with neither the L5 marker Ctip2 (Additional file 2: Figure S2B1-B3) nor the L4 marker ROR [42] (Additional file 2: Figure S2C1-C3), suggesting that the Zbtb20 expression in UL neurons is restricted to L2-L3 neurons. The expression pattern in ULs was preserved at P8 but almost disappeared at P12 (data not shown). In the early postnatal SVZ, Zbtb20 also maintained a strong expression in Nestin + RGCs (Fig. 1h1-h3; 90 out of 92 assayed Zbtb20^+^ cells in SVZ were co-labeled for Nestin, 92 %, n = 3), suggesting a possible involvement in postnatal neurogenesis.

In summary, beginning at E11.5 in VZ of VP/LP, the expression of TF *Zbtb20* expands into the VZ of the entire pallium at E14.5 and thereafter, suggesting that the timed expression of Zbtb20 may be involved in generation and/or specification of the UL neurons.

### Disturbances in superficial neocortical layers in *Zbtb20* loss-of-function

**Fig. 2:**
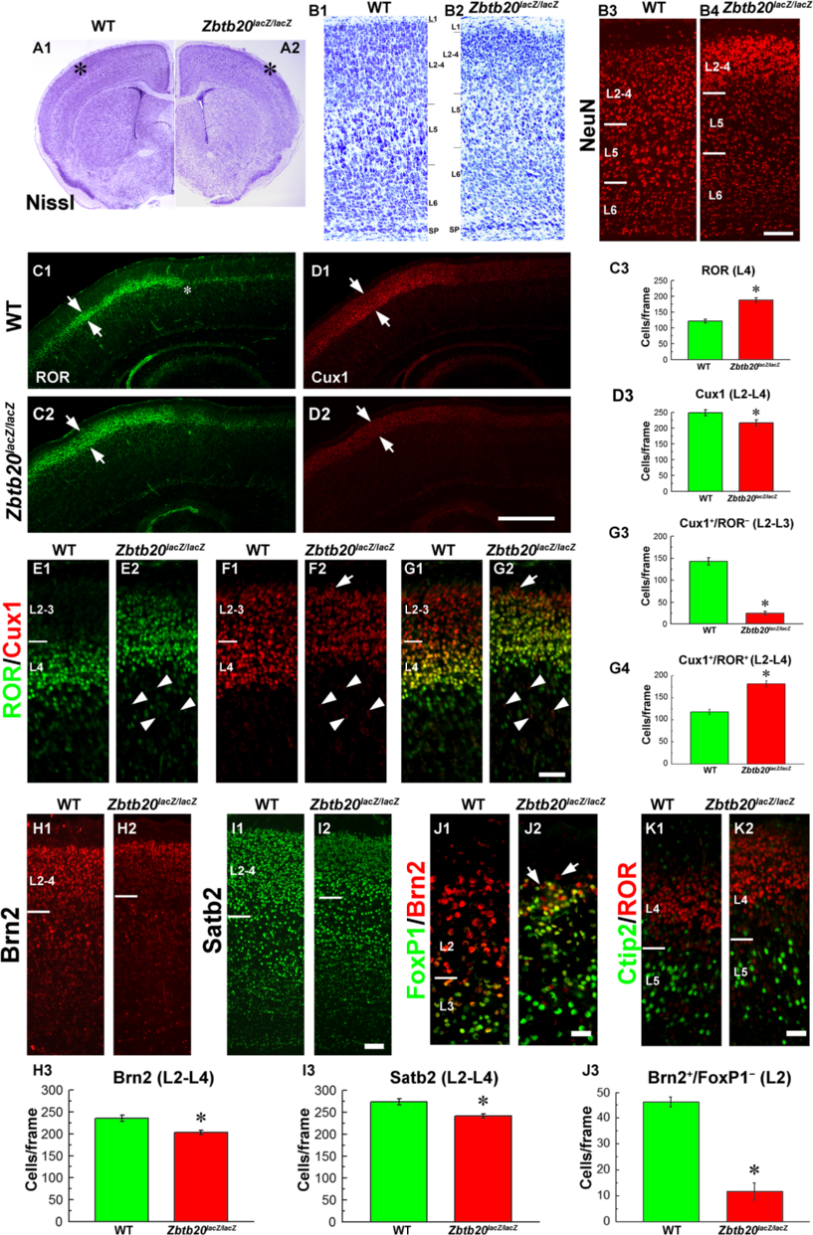
Upper layer defects in *Zbtb20* ^*lacZ/lacZ*^ mice. **a1-b2** Cresyl violet staining of brain cross sections from P28 wild type (WT) and mutant cortex. (b1, b2) are images at high magnification from fields in the somatosensory cortex, labeled with * in A1/A2. (b3-b4) NeuN IHC at stage P4. **c1-g4** Evaluation of UL neuronal fate by using ROR and Cux1 antibodies as markers, and a statistical analysis. In (c1-c2) note the expansion of the ROR signal (*arrows*), while in (d1-d2), a slight reduction of the Cux1-positive band (*arrows*) in the mutant Ncx. (e1-g2) are higher magnifications of SS cortex, labeled with the same markers. (c3,d3,g3,g4) represent graphs of statistical analysis of the number of positive cells/frame in SS cortex (*, *P* < 0.05, *n* = 3 *per* genotype). **h1-i2** IHC with Brn2 and Satb2 antibodies as UL markers on cross sections from P4 brains, and a statistical analysis of the number of stained cells. Note the reduction of the ULs in the mutant, which was statistically confirmed (*, *P* < 0.05, *n* = 3 *per* genotype). **j1-j3** Double IHC with Brn2 and FoxP1 antibody to evaluate UL neurons with L2 identity (Brn2^+^/FoxP1^−^). Note the drastic diminishing of the number of L2 neurons in the mutant Ncx (*arrows*), which is supported by a statistical analysis (*, *P* < 0.05, *n* = 3 *per* genotype). **k1-k2** Double immunostaining for ROR (L4) and Ctip2 (L5) reveals a preserved molecular border between the two layers in the mutant. Countings of positive cells were performed in frames sized 300 μm (h) × 100 μm (w) spanning the ULs of SS cortex. Scale bars: **b4**, 100 μm; **d2**, 500 μm; **g2**/**j2**, 50 μm; **i2**/**k2**, 100 μm

Cresyl violet (Nissl) staining of P10 coronal brain sections revealed apparent defects in cortical layering in *Zbtb20*^*lacZ/lacZ*^ mice (Fig. 2a1-b2). While the LLs (L6, L5) appeared overrepresented in the *Zbtb20*^*lacZ/lacZ*^ somatosensory (SS) cortex, the ULs (L4-L2) seemed thinner (Fig. 2b1-b2). This impression was confirmed by NeuN immunoassaying (Fig. 2b3-b4). In order to investigate the UL disturbances in more detail, we performed immunostaining for specific UL neuronal markers. Given the widespread expression of Zbtb20 in VZ of the entire pallium at E14.5, we first investigated the expression of the L4 marker ROR [43]. Intriguingly, in the mutant cortex, the thickness of L4 was greatly augmented (Fig. 2c1-c3). This notion was confirmed by another marker with a strong expression in L4, TF *CoupTF1* (see below). Immunoassaying for the global (L2-L4) UL marker Cux1 [18, 19], however, showed a reduction of UL neurons (Fig. 2d1-d3). Additional UL markers, Brn2 (L2, L3, and L5; [40, 41]) and Satb2 (L4-L2; [7, 8]) confirmed the strongly diminished representation of UL subsets in the *Zbtb20*^*lacZ/lacZ*^ mice (Fig. 2h1-i3).

The overall reduction of ULs, accompanied by the selective expansion of L4, raised a question on the status of the L2-L3 neurons in the mutant cortex. Therefore, we quantified the L3-L2 Cux1^+^/ROR^−^ population (Fig. 2f2, g2, arrow), which was markedly depleted (Fig. 2g3), in contrast to the L4 Cux1^+^/ROR^+^ population, which was augmented (Fig. 2g4). In order to study whether L3 or L2 neuronal subsets were specifically affected, we made use of the expression patterns of TF FoxP1, which is expressed in L6a and L5-L3 [44] and TF Brn2, which specifically marks L3 and L2 [40, 41]. Double immunostaining for FoxP1 and Brn2 revealed an ectopic expansion of FoxP1 expression into the normal position of L2 in the mutant cortex, while cells specifically fated to L2 identity (Brn2^+^/FoxP1^−^, Fig. 2j1-j2, arrows) were almost completely missing (Fig. 2j3). The depletion of the ULs was not due to an enhanced apoptosis, as studied by the expression of activated Caspase-3 (data not shown). To investigate whether the molecular boundary between L5 and L4 was preserved, we applied Ctip2 (L5)/ROR (L4) double immunohistochemical (IHC) staining, and we found that these two subpopulations of cortical neurons were properly segregated (Fig. 2k1-k2).

To sum up, these findings indicate that Zbtb20 deficiency results in a significant diminishing of L3 and especially L2 neuronal subsets as well as in an augmented and ectopic presence of L4 neurons in UL position.

### Enhanced deep layers and normal arealization in *Zbtb20*^*lacZ/lacZ*^ cortex

**Fig. 3:**
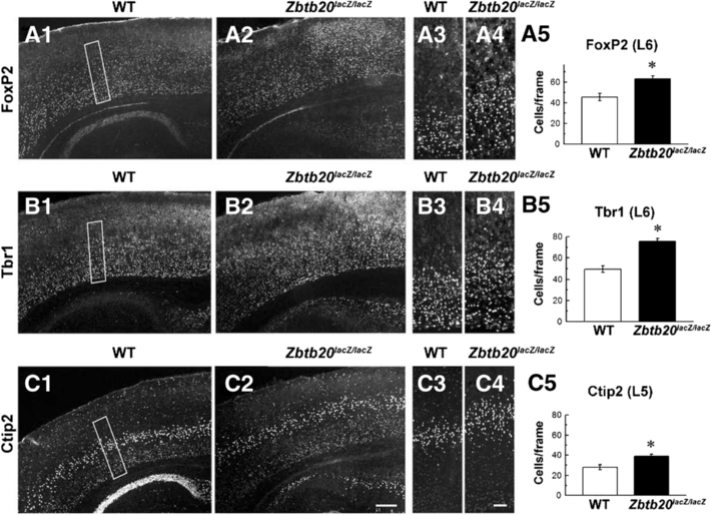
Enhanced presentation of LL neurons in somatosensory cortex of *Zbtb20* ^*lacZ/lacZ*^ mutants. **a1-b4** IHC with FoxP2 (a1 –a5) and Tbr1 (b1-b5) antibodies as L6 markers reveals an increased number of L6 neurons in the mutant compared with WT cortex. **c1-c5** IHC with antibody Ctip2 as L5 marker shows an enhanced number of L5 neurons in the mutant. (a5,b5,c5) Graphs representing statistical evaluation of the results (*, *P* < 0.05, *n* = 3 *per* genotype). All stainings were performed at stage P4. Countings of positive cells were performed in frames sized 300 μm (h) × 100 μm (w) spanning the LLs of SS cortex. Scale bars: **c2**, 200 μm, **c4**, 100 μm

To study quantitatively the apparent enhancement of both L5 and L6 sets in the mutants (Fig. 2b1-b4), we performed IHC staining and counting of cells in the SS cortex on cross sections of both genotypes with antibodies for TFs FoxP2 [44] in L6 (Fig. 3a1-a4), Tbr1 [9] in L6 (Fig. 3b1-b4) and Ctip2 [15, 45] in L5 (Fig. 3c1-c4). Indeed, the results revealed a statistically significant increased number of both L6 and L5 neurons in the mutant as compared with the control Ncx (Fig. 3a5, b5, c5). Furthermore, ISH staining of sagittal P4 brain sections indicated that the mutant cortex displayed enhanced LL neuronal subsets, including *Fezf2*^+^ L5 [15, 46] (Additional file 3: Figure S3A1-A4), *Clim1*^+^ L5 [46] (Additional file 3: Figure S3B1-B4), and *Id2*^+^ L5 and *Id2*^+^ L6 [47] (Additional file 3: Figure S3C1-C4). In order to confirm that the described layering abnormalities in the *Zbtb20*^*lacZ/lacZ*^ mice are not restricted to the SS cortex, we studied the patterning of the primary motor cortex and, similarly to SS cortex, we found a decrease of the ULs and enhancement of the LLs (Additional file 4: Figure S4).

In layer 5**,** the rostral limit of expression of TF *Id2* outlines the position of the border between the motor/somatosensory (M/SS) cortex, which is also marked by the caudal limit of *Id2* expression in L3-L2 [48]. In *Zbtb20*^*lacZ/lacZ*^ cortex, the M/SS boundary appeared rostrally displaced into the M field (Additional file 3: Figure S3C1-C2, asterisk). We therefore examined other cortical area-specific markers, including *Cadherin-8* (Additional file 3: Figure S3D1-D2, asterisk), Serotonin (Additional file 3: Figure S3E1-E2, asterisk), Bhlhb5 (Additional file 3: Figure S3 F1-F2, asterisk), ROR (Fig. 2c1-c2, asterisk, and data not shown), *Cadherin-6* and *Lmo3* (data not shown). None of these markers exhibited shifts in the mutants along the antero-posterior axis, so we concluded that the neocortical arealization in *Zbtb20*^*lacZ/lacZ*^ mice is grossly not affected. This notion is also supported by the preserved pattern of the graded expression of TFs *Pax6, Emx2, Foxg1* and *Lhx2* in *Zbtb20*^*lacZ/lacZ*^ embryonic pallium at E12.5 [35], a stage at which these TFs are known to have crucial roles in specification of the intrinsic program of cortical arealization encoded in the progenitors [23].

### ZBTB20 modulates the temporal onset for generation of distinct neuronal layer identities

**Fig. 4:**
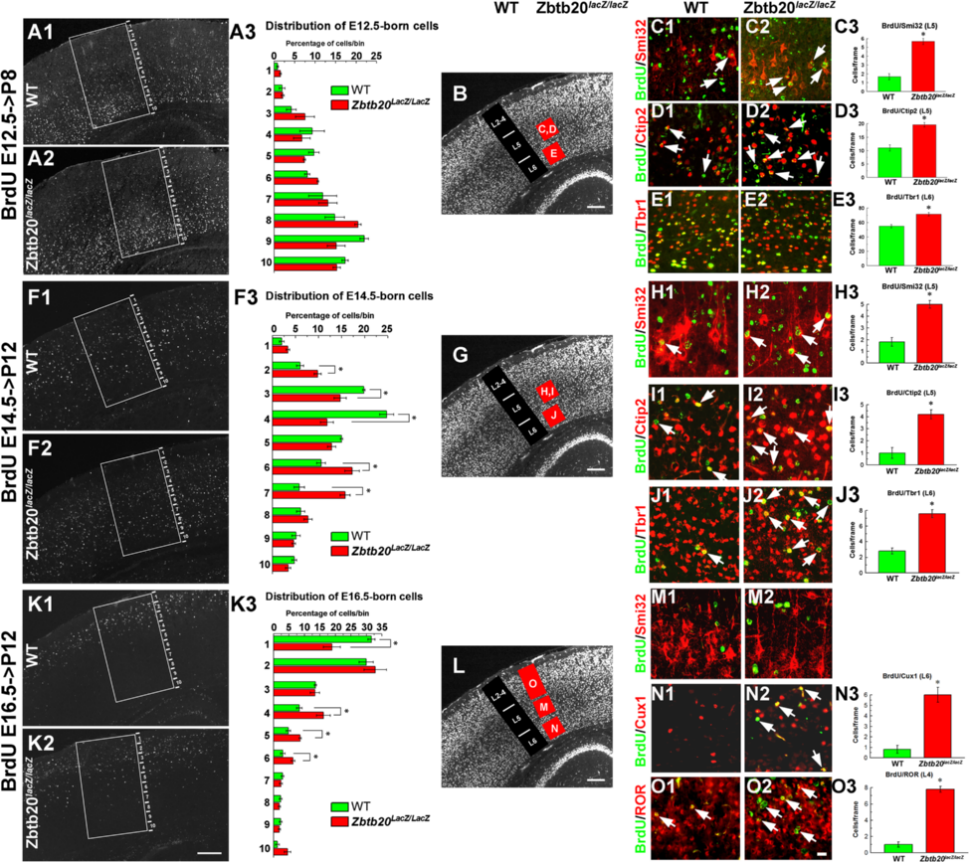
Laminar distribution and fate of neurons born at E12.5, E14.5 and E16.5 in WT and *Zbtb20* ^*lacZ/lacZ*^ neocortex. BrdU was injected at each of the above indicated embryonic stages and the distribution of the BrdU-tagged cells was evaluated in the postnatal SS cortex. **a1-e3** Distribution and fate of cells born at E12.5 and investigated at P8. (a1-a3) Overview images of the localization of BrdU^+^ cells to bins 1–10 and a statistical analysis showing no significant differences between WT and mutant in any of the bins. **b** NeuN IHC providing an overview of the cortical layers and the position of frames where counting was done in c1-e3 (red squares with respective letters). (c1-e2) Cell fate of E12.5-generated neurons in L5 (co-stained for Neurofilament-H/Smi32 and Ctip2) and L6 (co-labeled for Tbr1), and a statistical analysis (c3,d3,e3, *, *P* < 0.05, *n* = 3 *per* genotype). Double-labeled cells are depicted by arrows. **f1-j3** Distribution and fate of cells born at E14.5 and investigated at P12. (f1-f3) Overview images of the localization of BrdU^+^ cells to bins 1–10 and a statistical analysis showing a significant increase of the proportion of BrdU^+^ cells distributed in the lower bins (6–7), and a reduction of cells localized in upper bins (3–4) in the mutant cortex as compared to WT. (g) NeuN IHC providing an overview of the cortical layers and the position of frames where counting was done in h1-j2 (red squares with respective letters). (h1-j3) Fate of E14.5-generated neurons with L5 identity (co-stained for Neurofilament-H/Smi32 and Ctip2) and L6 (co-labeled for Tbr1), and a statistical analysis (H3,I3,J3, *, *P* < 0.05, *n* = 3 *per* genotype). Double-labeled cells are depicted by arrows. **k1-o3** Distribution and fate of cells born on E16.5 in P12 SS cortex. (k1-k3) Overview images of the localization of BrdU^+^ cells to bins 1–10 and a statistical analysis showing a significant increase of the proportion of BrdU^+^ cells localized in bins 4–6 in mutant, while a reduction of the percentage of cells localized in uppermost position in bin 1 as compared to WT. (l) NeuN IHC providing an overview of the cortical layers and the position of frames where counting was done in m1-o2 (red squares with respective letters). (m1-o3) Fate of E16.5-generated neurons. (m1-m2) In L5, none of the BrdU+ cells in either WT or mutant co-expressed Neurofilament-H/Smi32. (n1-n3) Co-labelling of BrdU+ cells with UL marker Cux1 in L6 shows an increased number of retained in the LLs Cux1^+^ cells most of which express BrdU (*arrows*). (n3) Statistical analysis (*, *P* < 0.05, *n* = 3 *per* genotype). (o1-o3) Double-labelling for BrdU and ROR demonstrates the strongly increased numbers of E16.5-born L4 ROR^+^ cells in the mutant compared to WT cortex (*arrows*). (n3) Statistical analysis (*, *P* < 0.05, *n* = 3 *per* genotype). All countings of the laminar distribution of BrdU^+^ cells (A1/A2,F1/F2,K1/K2) were performed within frames sized 800 μm (h) × 400 μm (w) spanning the entire cortical thickness, subdivided into 10 equally-sized bins. BrdU/Ctip2 and BrdU/NF colabaling was evaluated in 200 μm (h) × 200 μm (w) frames, while BrdU/Tbr1, BrdU/Cux1 and BrdU/ROR co-staining was evaluated within 300 μm (h) × 100 μm (w) frames, positioned as presented in panels B/G/L. **b**/**g**/**l** depict NeuN immunostaining of a WT cortex. Scale bars: **k2**/**b**/**g**/**l**, 200 μm, **o2**, 20 μm

To investigate whether Zbtb20 controls the switch to generation of neurons with different layer identities, we performed BrdU birthdating experiments at E12.5, E14.5 and E16.5 when predominantly LLs (L6, L5), L4 or L3-L2 neurons are born, respectively (Fig. 4). Taking advantage of the fact the pattern of NeuN immunostaining allowed distinguishing the location of LL and UL in the postnatal cortex (Fig. 2b3-b4), we calculated the percentage of BrdU^+^/NeuN^+^ cells located to either LLs (L5-L6) or ULs (L2-L4) out of the total BrdU^+^/NeuN^+^ cells in frames spanning the entire cortex (Additional file 5: Figure S5). Analysis of the position of the E12.5-born cells revealed no differences in their laminar location between WT and the mutant (Fig. 4a1-a3; Additional file 5: Figure S5A1-A3). In contrast, birthdating at E14.5, revealed a significantly larger proportion of BrdU^+^ cells in the deep position of the *Zbtb20*^*lacZ/lacZ*^ cortex, and on contrary, a smaller fraction of such cells was found in the superficial position of the mutants (Additional file 5: Figure S5B1-B3; also Fig. 4f1-f3). Similarly, BrdU pulse labelling at E16.5 (at the peak of UL generation) showed a reduced distribution of tagged cells at superficial position and significantly more deeply located BrdU^+^ cells in the mutant cortex (bins 4–6; Fig. 4k1-k3), that was also evident on BrdU/NeuN double-stained sections (Additional file 5: Figure S5C1-C3).

Using BrdU co-staining with layer-specific neuronal markers, for L5 (Neurofilament/Smi32 and Ctip2) and L6 (Tbr1), we quantitatively investigated the layer fate of cells generated at stages E12.5, E14.5 or E16.5**.** The results revealed that the mutant Ncx had significantly increased populations of L6 and L5 neurons born at E12.5 (Fig. 4c1-e3), as well as at E14.5, the peak of generation of L4 neurons during normal corticogenesis (Fig. 4h1-j2). At E16.5 such expansion of L6/L5 fate identities was no more evident (Fig. 4m1-m2). However, the cells born at E16.5 in the mutant Ncx demonstrated a significant increase of the co-labeling with the L4 marker ROR as compared to the control Ncx (Fig. 4o1-o2, arrows, O3). These results suggest that the developmental window for generation of L6-L5-L4 neurons was expanded by at least 2 days for each neuronal type, which will profoundly affects the progenitor pool size for L3-L2 neurons. In a further support of such a scenario were the results after analysis of the fractions of Cux1^+^/ROR^+^ (L4) and Cux1^+^/ROR^−^ (L3-L2) neuronal subsets born at stages E12.5, E14.5 or E16.5 in WT and mutant mice (Additional file 6: Figure S6). We found that at P12, the UL fractions born at E12.5 or E14.5 did not differ significantly between WT and mutant mice (Additional file 6: Figure S6A1-B3). However, changes were observed in the E16.5-born UL neuronal fractions in *Zbtb20* LOF as compared to WT: the L4 fraction was larger, while the L3-L2 fraction was diminished (Additional file 6: Figure S6C1-C3).

Together, these results strongly suggest that the timed expression of transcriptional repressor *Zbtb20* in cortical progenitors (appearing in the entire pallium only after E14.5) could control the transition from early- versus late born neuronal layer identities.

### Impaired late neurogenesis and neuronal migration in *Zbtb20*-deficient cortex

**Fig. 5:**
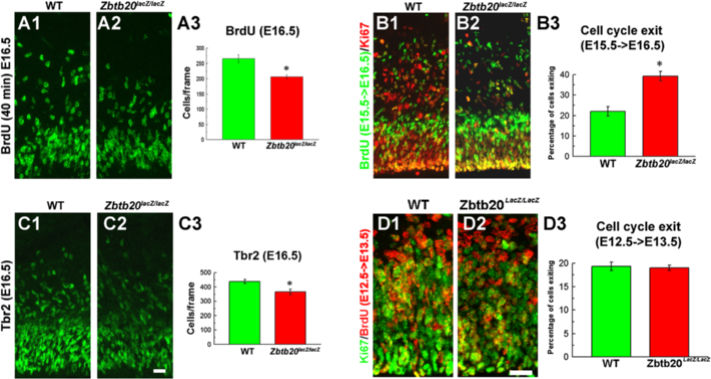
Altered cell cycle parameters in *Zbtb20* ^*lacZ/lacZ*^ mice. **a1-c3** Impaired cell cycle kinetics in DP of *Zbtb20* mutant during late neurogenesis at stage E16.5. **a1-a3** Decreased proliferation as measured by short - term (40 min) BrdU pulse labelling. Cells were evaluated in frames sized 300 μm (h) × 200 μm (w) in DP. **b1-b3** Co-immunostaining for BrdU and Ki67 after 24 h BrdU pulse labelling (E15.5- > E16.5) revealed an increased percentage of cells exiting the cell cycle in the DP of the mutant as compared with the WT cortex. Cells were evaluated in frames sized 300 μm (h) × 100 μm (w) in DP. **c1-c3** Labelling with Tbr2 antibody indicates a depletion of intermediate progenitors in *Zbtb20* ^*lacZ/lacZ*^ DP. Cells were evaluated in frames sized 300 μm (h) × 200 μm (w) in DP. Statistical analysis (a3,b3,c3) proves that the differences are significant (*, *P* < 0.05, *n* = 3 *per* genotype). **d1-d3** Unaltered cell cycle exit kinetics for early (BrdU-tagged at E12.5) progenitors at E13.5. Cells were evaluated in frames sized 150 μm (h) × 75 μm (w) in DP. No change in the percentage of cells exiting the cell cycle in the mutant DP as calculated by BrdU/Ki67 co-staining after a 24 h (E12.5- > E13.5) BrdU pulse (*P* > 0.05, *n* = 3 *per* genotype). Scale bars: 20 μm

By using 40 min BrdU-pulse labelling in vivo at E16.5 (the peak of production of UL neurons) we found a significant reduction of the progenitor proliferation in the *Zbtb20LOF* cortex (Fig. 5a1-a3). Additionally, BrdU/Ki67 double-labelling after a 24 h BrdU pulse (E15.5- > E16.5), indicated an increased progenitor exit from the mitotic cycle as measured by the percentage of the BrdU^+^/Ki67^−^ cells versus all BrdU^+^ cells (Fig. 5b1-b3). Consistent with these data, we found at E16.5 in the mutant DP a reduction of Tbr2^+^ IPs (Fig. 5c1-c3), the main neuronal source for generation of neurons with an UL neural fate [49]. Notably, we did not detect changes in the progenitor cell exit during early neurogenesis (time window of LL generation) using a 24 h BrdU pulse at E12.5- > E13.5, followed by BrdU/Ki67 double-labelling (Fig. 5d1-d3).

Our previous expression analysis of Zbtb20 in developing cortex at stage E18.5 suggested a migratory delay of NeuroD1^+^ neurons (Fig. 4h2 of [35]). At the same stage, we showed here a band of Id2^+^, Math2/Nex^+^ neurons in the pallial SVZ in *Zbtb20 KO* mice (Additional file 7: Figure S7, arrows), suggesting migratory abnormalities of the lately-born neurons. Indeed, in the UL birthdating experiments at E16.5, the mutant Ncx contained significantly more E16.5-born BrdU^+^/Cux1^+^ cells in the L6 *(*Fig. 4n1-n3) as well as in the subcortical white matter (data not shown). Notably, the retained Cux1^+^ cells were ROR^−^ (Fig. 2e2/f2; arrowheads) indicating that a sub-population of correctly specified L3/L2 neurons exhibits an impaired migration towards their final destination to the CP. Notably, the postmitotic expression of Zbtb20 in cortical plate is confined to the Cux1^+^/ROR^−^ population.

### *Zbtb20* deficiency affects the expression of *CoupTF1* in developing cortex

**Fig. 6:**
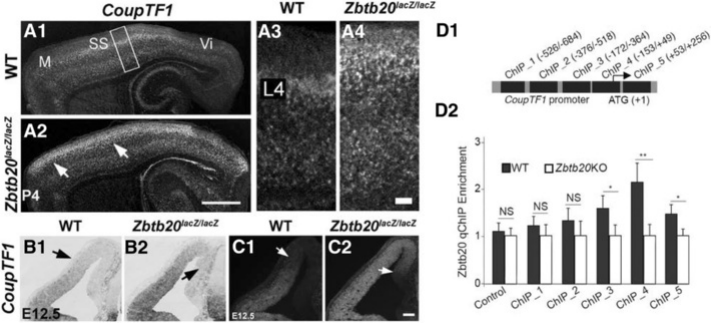
Regulation of *Coup-TF1* expression by *Zbtb20*. **a1-c2** Striking enhancement of the expression of TF *CoupTF1* in *Zbtb20* ^*lacZ/lacZ*^ mutants. (a1-a4) Ectopic expansion of the *CoupTF1* signal in the SS area at P4 as detected by ISH. Note the shift of a strong *CoupTF1* signal in a wide band in the ULs of the mutant (*arrows* in a2). (b1-c2) Ectopic expansion of *CoupTF1* expression in mutant DP/MP at stage E12.5, detected either by ISH (b1-b2) or IHC (c1-c2). **d1-d2** Zbtb20 binds to the *CoupTF1* promoter. **d1** The scheme depicts relative positions of fragments on the promoter and the first intron of *CoupTF1* that are used in ChIP experiment. **d2** ChIP analysis for the *CoupTF1* promoter and first intron occupancy by Zbtb20 in cortical neural stem cells from wild type (WT) and *Zbtb20* ^*lacZ/lacZ*^ cortices (as negative control). Scale bars: **a2**, 1 mm; **a4**/**c2**, 100 μm

As noticed, the orphan nuclear receptor *CoupTF1*, whose strong expression is normally restricted to L4 in the SS area [45, 50] was ectopically expressed as a thick band along the entire AP axis of the *Zbtb20* mutant (Fig. 6a1-a4; arrows). Similar to the presented here abnormalities of Ncx in *Zbtb20* LOF, overexpression of *CoupTF1* in the pallial VZ promotes progenitor exit from mitotic cycle, inhibits the IPs production and causes enhanced generation of early-born at the expense of late generated neuronal fates [51]. At E12.5, both CoupTF1 [50, 52] and Zbtb20 are expressed at the corticostriatal border in faint DV gradients. Indeed, the double IHC at E12.5 showed a co-localization of Zbtb20 and CoupTF1 in the VZ of the control animals (Fig. 1c1-c3), and significant enhancement of CoupTF1 expression in *Zbtb20*^*lacZ/lacZ*^ mice as revealed by both ISH (Fig. 6b1-b2) and IHC (Fig. 6C1-C2*)*. Thus, the observed increased generation of early-born neuronal fates (L6,L5) in *Zbtb20 LOF* might be mediated by modulation of *CoupTF1* expression.

To study a possible regulation of *CoupTF1* expression by *Zbtb20* at molecular level, we investigated whether Zbtb20 binds to the promoter of *CoupTF1* [53] (Fig. 6d-d2, Additional file 8: Figure S8). In ChIP assays, we used neural stem cultures (NSC) from E15.5 cortices from WT and *Zbtb20KO* embryos and Zbtb20 antibody. We found that Zbtb20 binds with low affinities with fragments at locations of −172/-364 (ChIP_3) and +53/+256 (ChIP_5) of the *CoupTF1* promoter and the first intron (Additional file 8: Figure S8). Zbtb20 occupies the *CoupTF1* promoter with highest affinity at the location of −153/+49 (ChIP_4), which contains multiple DNA binding motifs of Zbtb20 [33] (Fig. 6d1-d2, Additional file 8: Figure S8). These data suggests that TF *Zbtb20* is a regulator of the expression of *CoupTF1* most probably acting as a repressor. In a further support, ISH with *Zbtb20* in situ probe on E15.5 brain sections from WT and *CoupTF1*^*−/−*^ mice [51], showed a decreased mRNA signal (Additional file 9: Figure S9; arrows) suggesting a cross regulatory loop between these two TFs.

## Discussion

Here we identify TF Zbtb20 as essential regulator of the timed sequential generation of distinct neuronal layer identities in developing cortex. Our results suggest that by executing a robust expression in the germinative zone of the entire pallium after E14.5, the Zbtb20 imposes cell-intrinsic temporal limits for generation of L6-L4 versus L3-L2 neuronal fates.

### Zbtb20 is a regulator of timed neurogenesis in developing neocortex

**Fig. 7:**
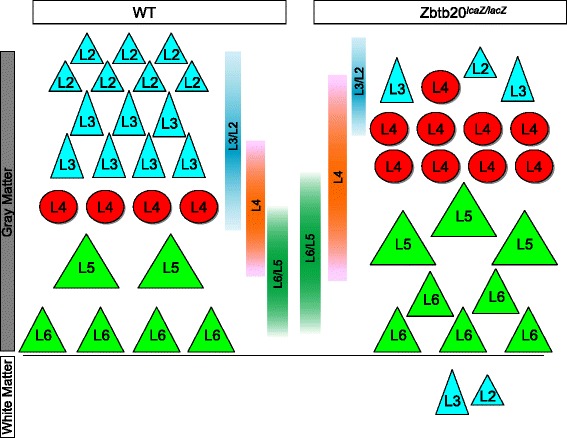
Schematic summary of the observed effects of Zbtb20 on the temporal acquisition of identity by the cortical glutamatergic projection neurons. **a** Arrangement of cortical pyramidal neurons in WT mice and their normal time windows of generation. **b** Layer defects in *Zbtb20* ^*lacZ/lacZ*^ mice and the respective changes in the time windows for neuronal generation. Note the increase of L6, L5 and L4 neurons and the diminishing of L3 and L2 neurons. A few ROR^+^ (L4) cells can even be seen intermingled with L3/L2 cells. Some L3/L2 cells are retained in the subcortical white matter and LLs

During corticogenesis, the timed generation of layer-specific fates depends on intrinsic and extrinsic cues acting at a given developmental stage [1]. This process includes consecutive steps of repression of cell fates generated during earlier stage. Here we showed that in a lack of TF Zbtb20 in the cortical progenitors, neurons with L6-L5-L4 identity continue to be produced heterochronicly, in an expanded by at least 2 days time window, thus substantially shortening the time for production of L3 and especially of L2 neurons (Fig. 7). We consider that this temporal alteration of the schedule for layer production together with the disclosed enhanced exit from mitosis of E16.5 progenitors are the main factors leading to L6-L5-L4 enlargement in the *Zbtb20LOF.* Because of its dynamics of expression, *Zbtb20* exerts different roles at distinct developmental stages: at E14.5, the time at which the full extent of *Zbtb20* expression in the pallial VZ progenitors is achieved, this TF restricts L6-L5 versus L4 fate, while at E16.5, the peak of UL generation, Zbtb20 restricts L4 versus L3-L2 fate. In addition, in *Zbtb20 LOF* cohorts of progenitors leave the cell cycle prematurely, diminishing the progenitor pool that remains for the latest-born neuronal types.

Genes known to regulate the sequential transitions between cortical subtypes include *CoupTF1* [51, 54, 55], *FoxG1* [14, 56], *Gli3* [57] and *Brn2* [58]. The expression of neither *FoxG1* [35] nor *Gli3* (this study, data not shown) were altered in *Zbtb20*^*lacZ/lacZ*^ mice. Interestingly, both Zbtb20 and CoupTF1 exerted a similar expression dynamics, starting in progenitors of VP, LP at E12.5 and progressively expanding at later stages in VZ/SVZ of the entire pallium, and even continue to be co-localized postnatally in UL neurons. As shown here, Zbtb20 binds the promoter of *CoupTF1*, and in *Zbtb20* LOF the expression of *CoupTF1* was elevated as early as E12.5, indicating that Zbtb20 controls directly the expression of *CoupTF1*. Notably, several aspects of the neocortical phenotype in *Zbtb20KO* mice are reminiscent to abnormalities observed after in vivo overexpression of *CoupTF1* [51], including: (*i*) expansion of early born neuronal sets, (*ii*) changed balance between early and late born neurons, (*iii*) enhanced cell cycle exit of progenitors and (*iv*) diminished production of IPs. On the contrary, in the pallium of *CoupTF1 KO* mice, the expression of Zbtb20 was reduced, making possible the existence of a feedback loop between *Zbtb20* and *CoupTF1*. The lack of birthdating and cell cycle abnormalities during L6-L5 neurogenesis in the mutant pallium during the early neurogenesis indicates that *Zbtb20* most probably exerts effects on temporal specification of the LLs via the ectopic *CoupTF1* expression.

Acting at the border between the archi- and isocortex, *Zbtb20* repression seems to be important to inhibit neocortical cell fate [33]. Upon overexpression in progenitors of medial pallium, Zbtb20 directly binds and represses the activity of genes that are specifically expressed in LL neurons (*Tbr1, FoxP2, Fezf2, Ctip2, Sox5*) or ULs (e.g. *Rorb, Satb2, Cux1/Cux2*) [33]. However, in developing Ncx, Zbtb20 co-exists with, and thus might directly repress, only few of those TFs: *Fezf2* and *FoxP2* (in mitotic VZ precursors), *Cux1/2* (in mitotic cells in VZ/SVZ as well as in postmitotic cells in ULs), and *Satb2/Brn2* (in postmitotic ULs). The enhancement of markers which do not co-exist in the same cells with Zbtb20, such as Ctip2 (L6 and L6) and ROR (L4), can be explained by action of *Zbtb20* at the level of the progenitors of these neurons. The expansion of Ctip2^+^ L5 subsets in *Zbtb20* LOF of neurons most probably involves a de-repression of the *Fezf2* gene in VZ/SVZ. Together with the results in [33], our data suggest that TF *Zbtb20* acts as a modulator of the *Fezf2/Ctip2/Tbr1/Satb2* network mostly in pallial VZ progenitors.

The mutant phenotype of the latest-born Zbtb20^+^ L3-L2 cells suggests also an involvement of *Zbtb20* at the postmitotic level, probably influencing their proper migration towards the cortical plate**.** The involvement of Zbtb20 in the regulation of neuronal migration of late-born UL cells was supported by our findings indicating: (i) retention of Cux1^+^/ROR^−^ (Fig. 2f2, arrowheads) and Bhlhb5^+^ (Additional file 3: Figure S3 F2, inset) cells below the ULs of the mutant at P12; (ii) their birth date at E16.5 (Fig. 4n2); (iii) accumulation of postmitotic cells in the mutant SVZ at E18.5 (Additional file 7: Figure S7). Thus, the prenatal reduction of UL precursor pool is possibly exacerbated by a postnatal migrational deficit of L3-L2 neurons. Potential target genes for this deficit are TFs Cux1 which is expressed in both mitotic and postmitotic progenitors [19], and Brn2 [40] which was recently shown as a possible Zbtb20 downstream target [59].

## Conclusions

Transcription factor *Zbtb20* is expressed in the ventricular zone of the developing neocortex in a dynamic spatiotemporal pattern. Loss of *Zbtb20* in cortical radial glia cells leads to prolongation of the developmental time limits for sequential production of early-born cortical layer identities (L6, L5 and L4). This dramatically shortens the time of production of later born (L3, L2) neuronal sets which are severely underrepresented. Mechanistically, the deficiency of Zbtb20 leads to decreased proliferation and enhanced exit from mitotic cycle of the late cortical progenitors, an effect mediated at least in part via modulation of the expression of *CoupTF1/Nr2f1*.

## Methods

### Animal experiments

Animals were handled in accordance with the German Animal Protection Law, after an approval of the experiments by the Niedersächsische Landesamt für Verbraucherschutz und Lebesmittelsicherheit (LAVES)/Oldenburg, contract No 33.9-42502-04-11/0622 from 07.12.2011. Experiments were completed prior to January 1^st^ 2013. All surgical procedures were performed under isoflurane/N_2_O anesthesia, and all efforts were made to minimize suffering. The *Zbtb20* gene targeting and the generation of the transgenic mice have been previously described [35]. The *Zbtb20* knock out (KO) mice lack the functionally important BTB/POZ domain of the protein as well as the first of five zinc fingers, which were replaced by a *lacZ-neomycin* cassette. Therefore, homozygous mutants will be referred to as *Zbtb20*^*lacZ/lacZ*^ mice within this study. The specificity of the deletion and the complete loss of Zbtb20 protein have been confirmed as described [35].

### ChIP analysis

ChIP analyses were performed as described previously [60] with some modifications. Cortical progenitors were prepared from E15.5 WT and *Zbtb20KO* mice [35] and cultured as described [61]. ChIP experiments were performed using an EZ ChIP assay kit (Millipore), according to the supplier’s instructions with Zbtb20 antibody (HPA016815, Sigma). A genomic fragment of the *Trim11* gene and a GFP antibody plus IgG were used as negative DNA and antibody controls, respectively [60]. Primer sequences are listed Additional file 10: Table S1.

### Histological processing

Isolated embryos or brains at defined stages were washed in cold phosphate-buffered saline (PBS) and fixed in 4 % paraformaldehyde (PFA) overnight at 4 °C. Tissues were rinsed in PBS, and processed for standard cryo-embedding. Cryosections (16 μm thick) were washed and blocked for 1 h in blocking solution containing a normal serum. Primary antibodies were incubated overnight at 4 °C in the blocking solution. After washing, the sections were incubated with species-specific secondary antibodies from the Alexa series (Invitrogen) in blocking solution for 2 h at room temperature (RT), washed again and mounted with Vectashield mounting-medium (Vector Labs) containing DAPI. We used the following primary antibodies/dilutions: mouse anti-β-galactosidase (1:200; Promega, Madison, WI), rat anti-BrdU (1:200; Abcam), mouse anti-BrdU (1:50; BD Bioscience), goat anti-Brn2 (1:50, Santa Cruz Biotech, Santa Cruz, CA), rabbit anti-Caspase-3 (1:200; Cell Signalling, Cambridge, UK), mouse anti-CoupTF1 (1:1000; Perseus Proteomics, Tokyo, Japan), rat anti-Ctip2 (1:200; Abcam), rabbit anti-Cux1 (1:250; Santa Cruz), rabbit anti-FoxP1 (1:500, Abcam), rabbit anti-FoxP2 (1:500, Abcam), rat anti-Ki67 (1:100; Dako), mouse anti-Nestin (1:100; Millipore, Billerica, MA), mouse anti-NeuN (1:100; Millipore), mouse anti-Neurofilament (1:100; Abcam), rabbit anti-Pax6 (1:300; Covance), mouse anti-Pax6 (1:100; Developmental Studies Hybridoma Bank, DHSB), mouse anti-phospho-Histone H3 (1:50; Cell Signalling), mouse anti-phospho-Vimentin (1:300; MBL), mouse anti-ROR (1:100; Perseus Proteomics), mouse anti-Satb2 (1:200; Abcam), mouse anti-Sox2 (1:50; R&D systems), rabbit anti-Tbr1 (1:300; Abcam), rabbit anti-Tbr2 (1:200; Chemicon), rabbit anti-Zbtb20 (1:25–1:100, Sigma). The anti-BrdU antibodies were visualized after pre-treatment of tissues in 2 N HCl at 37 °C for 30 min. The anti-Zbtb20 antibody was used after an antigen retrieval by heating in a microwave (800 W, 3 times, 5 min each) in a citrate buffer (pH 6.0).

### In situ hybridization

Whole heads from E12.5 or whole brains from E18.5 or P4 mice were dissected in ice-cold DEPC-treated PBS, fixed in 4 % PFA/PBS for 3 h at 4 °C, washed in PBS, and incubated in 25 % sucrose overnight at 4 °C. Specimens were sectioned at 16 μm after embedding and freezing in OCT cryomatrix (Leica Microsystems Nussloch GmbH, Wetzlar, Germany). Nonradioactive in situ hybridization was done as described [27].

### Image analysis and quantification

Images were captured with an Olympus BX60 microscope, a Leica DM6000 epifluorescent system or a laser confocal microscope (Leica Sp5). For cell counts in sections from wild type (WT) and homozygous brains, we blindly counted the positive cells within equally sized frames (size of frames provided in the figure legends) on coded cross sections of somatosensory cortex in WT and mutant mice (n ≥ 3 *per* genotype). For BrdU birthdating experiments, frames spanning the entire cortex on cross brain sections were divided into 10 equally-sized bins, the BrdU^+^ cells in every bin were counted and divided by the total number of BrdU^+^ cells in all 10 bins. The size of the counting frames for BrdU/marker co-localization are provided in the figure legends.

Laser confocal microscopy was used to verify co-localization of multiple fluorescent signals. We performed Z sectioning at 0.5-1 μm intervals and optical stacks of at least 10 images were used for analysis, by the Leica Advanced Fluorescence software version 2.3.6. All images were processed with Adobe Photoshop (Version CS2) by overlaying the pictures, adjusting brightness, contrast and size.

### Statistical analysis

Statistical evaluation was performed by Student’s *T*-test or one-way ANOVA followed by Tukey-Kramer’s *post hoc* analysis. Statistical significance between control and experimental condition was considered if *p* < 0.05. Data are presented as means ± s.e.m.

## Abbreviations

CP, cortical plate; CSMN, corticospinal motor neurons; DP, dorsal pallium; GOF, gain-of-function; Hi, hippocampus; LL, lower layer; LOF, loss-of-function; LP, lateral pallium; MZ, marginal zone; Ncx, neocortex; pVim, phosphorylated vimentin; RGCs, radial glial cells; SS, somatosensory; SVZ, subventricular zone; TCA, thalamocortical axons; TF, transcription factor; UL, upper layer; VP, ventral pallium; VZ, ventricular zone.

## Additional files

Additional file 1: Figure S1. Expression of TF Zbtb20 in developing pallium. (A) ß-gal staining of *Zbtb20* ^*lacZ+/−*^ embryos at E11.5. The arrow points to a ß-gal activity expanding from the subpallium into the VZ of LP, while DP remains negative. (B) ISH on sagittal E14.5 brain sections reveals *Zbtb20* expression in the entire pallial VZ (arrows). Expression in MZ is depicted by arrowheads. (C1-C2) ISH on cross E16.5 brain sections demonstrates a strong *Zbtb20* expression in the pallial VZ (asterisk), gradually decreasing in the SVZ (C2). Also evident is a positive signal in the lateral migratory stream (arrow) as well as in MZ (arrowheads). The image in C2 corresponds to the boxed area in C1. BG, basal ganglia; DG, dentate gyrus, DP, dorsal pallium; Hi, hippocampus; LP, lateral pallium; Ncx, neocortex; Str, striatum; MP, medial pallium, VP, ventral pallium. Scale bars: A, 100 μm; B/C1 500 μm; C2, 100 μm. (PDF 451 kb) Additional file 2: Figure S2. Expression of TF ZBTB20 in UL neurons in early postnatal stage (P4) neocortex. (A1-A3) Double immunostaining for Zbtb20 and Brn2, and an overlay on brain cross section. Arrows depict double-positive cells at uppermost position in the developing Ncx. (B1) Immunostaining for TF Ctip2 (counterstained for DAPI) on brain cross section marks L5. The frame corresponds to the position of images shown in B2-B4. (B2-B4) Few L5 neurons express Zbtb20. Co-staining for Zbtb20 and Ctip2, and an overlay on brain cross sections. Arrows depict Zbtb20^+^ cells in L5 which do not exhibit co-labeling for Ctip2. (C1) Immunostaining for nuclear receptor ROR (counterstained for DAPI) on brain cross section (the strong ROR immunosignal marks L4). The frame corresponds to the position of images shown in C2-C4. (C2-C4) Co-staining for Zbtb20 and ROR, and an overlay on brain cross sections. The Zbtb20^+^ neurons in the upper layers do not exhibit co-labeling for ROR (arrows). Scale bars: A3, 50 μm; B1/C1, 100 μm; B4/C4, 20 μm. (PDF 161 kb) Additional file 3: Figure S3. Cortical arealization in *Zbtb20* ^*lacZ/lacZ*^ mice. (A1-C4) ISH analysis on brain sagittal sections at matched levels revealing enhancement of L5 neuronal subsets, marked by *Fezf2* (A1-A4), *Clim1* (B1-B4) and *Id2* (C1-C4), in the *Zbtb20* ^*lacZ/lacZ*^ mutants. The pattern of *Id2* expression confirms the increase of both L6 and L5 neurons, accompanied by a drastic decrease of the ULs in the *Zbtb20KO* cortex at P4. Asterisk in C1/C2 points to the presumptive border between the motor (M) and somatosensory (SS) area which appears rostrally displaced in the mutant. Images in A3/A4, B3/B4 and C3/C4 correspond to a field in SS cortex (indicated by a frame in A1,B1,C1). (D1-F2) However, ISH staining for *Cadherin 8* (D1-D2) and IHC labelling for Serotonin (E1-E2) and Bhlhb5 (F1-F2) on sagittal sections indicates a normal position of the M/SS border (asterisk). Scale bar: **C2**, 1 mm. (PDF 176 kb) Additional file 4: Figure S4. Enhanced presence of lower layer neurons in primary motor cortex of *Zbtb20* ^*lacZ/lacZ*^ mutants. (A1-A2) NeuN IHC demonstrating an overview of the motor cortex (depicted on the scheme in the upper left corner) on cross brain sections. The enlargement of L6 and L5 and the thinning of L2-L4 is apparent. (B1-B3) Decreased numbers of L2-L4 Cux1-immunostained neurons in *Zbtb20*-deficient cortex. (C1-D3) Immunostaining with FoxP1 (C1,C2) and Ctip2 (D1,D2) antibodies revealed in the Zbtb20 mutant an increased number of both FoxP2^+^ L6 and Ctip2^+^ L5 neurons. (B3,C3,D3) Graphs representing statistical evaluation of the results (*, *P* < 0.05, *n* = 3 *per* genotype). All stainings were performed at stage P8. Countings were performed within frames sized 400 μm (h) × 300 μm (w) for FoxP2, 300 μm (h) × 300 μm (w) for Ctip2, 500 μm (h) × 300 μm (w) for Cux1. Scale bars: A2, 200 μm, B2/C2/D2, 100 μm. (PDF 183 kb) Additional file 5: Figure S5. Distribution of neurons born at E12.5, E14.5 and E16.5 in WT and *Zbtb20* ^*lacZ/lacZ*^ mice across neocortical layers. BrdU was injected at each of the above pointed embryonic stages and the neocortical layers were visualized by NeuN IHC. We then calculated the percentage of BrdU^+^/NeuN^+^ cells in L6, L5 and L2-L4 out of the total BrdU^+^/NeuN^+^ cells in a frame 800 μm (h) × 200 μm (w) spanning through L2-L6. The image on the left side of the figure depicts NeuN immunostaining of a WT cortex (identical with Fig. 4b,g,l) providing an overview of the cortical layers. (A1-A3) BrdU (E12.5- > P8)/NeuN double IHC demonstrates the predominant distribution of BrdU^+^ cells in the deep (L5-L6) layers. No significant differences between the WT and mutant mice were detected (A3, *P* > 0.05, *n* = 3 *per* genotype). (B1-B3) BrdU (E14.5- > P12)/NeuN double IHC depicts a larger proportion double-positive cells in deep (L5-L6) layers of the mutant cortex (arrows in B2), while a lower percentage in the superficial (L2-L4) layers (B3, *, *P* < 0.05, *n* = 3 *per* genotype). (C1-C3) BrdU (E16.5- > P12)/NeuN double IHC reveals a larger proportion double-positive cells in mutant deep (L5-L6) layers (arrows in C2), while a lower percentage in the superficial (L2-L4) layers (C3, *, *P* < 0.05, *n* = 3 *per* genotype). LL, lower neocortical layers; UL, upper neocortical layers. Scale bar: C2, 50 μm. (PDF 139 kb) Additional file 6: Figure S6. Birth date analysis of specific subpopulations of neurons within the superficial (L2-L4) neocortical layers of WT and *Zbtb20* ^*lacZ/lacZ*^ mice. BrdU was injected at E12.5, E14.5 and E16.5 and BrdU IHC was combined with Cux1 and ROR immunostaining to distinguish between L4 (Cux1^+^/ROR^+^) and L2-L3 (Cux1^+^/ROR^−^) neuronal subsets. The BrdU^+^/Cux1^+^/ROR^+^ and the BrdU^+^/Cux1^+^/ROR^−^ neuronal subsets were calculated as a percentage of the BrdU^+^ cells within frames located in the upper layers within SS cortex. The same animals and corresponding frames were used as for Fig. 4 and Additional file 5: Figure S5. (A1-A3) Analysis of UL phenotypes born at stage E12.5. BrdU^+^/Cux1^+^/ROR^+^ cells are depicted by arrows, while the BrdU^+^/Cux1^+^/ROR^−^ cells - by arrowheads. No significant differences in the proportions of Cux1^+^/ROR^+^ (L4) and Cux1^+^/ROR^−^ (L2-L3) cells, born at this stage, were observed between the WT and the mutants (A3, *P* > 0.05, *n* = 3 *per* genotype). (B1-B3) Analysis of UL phenotypes born at stage E14.5. BrdU^+^/Cux1^+^/ROR^+^ cells are depicted by arrows, while the BrdU^+^/Cux1^+^/ROR^−^ cells - by arrowheads. Similarly to stage E12.5, no significant differences in the proportions of Cux1^+^/ROR^+^ and Cux1^+^/ROR^−^ cells, born at E14.5, were observed between the WT and the mutants (A3, *P* > 0.05, *n* = 3 *per* genotype). (C1-C3) Analysis of UL phenotypes born at stage E16.5. BrdU^+^/Cux1^+^/ROR^+^ cells are depicted by arrows, while the BrdU^+^/Cux1^+^/ROR^−^ cells - by arrowheads. Note that a significantly larger proportion of Cux1^+^/ROR^+^ (L4) cells are born at stage E16.5 in the mutant ULs than in the WT ULs (C3, *, *P* < 0.05, *n* = 3 *per* genotype). At the same time, in the *Zbtb20* deficient cortex, a significantly smaller percentage of BrdU^+^ cells showed L2-L3 (Cux1^+^/ROR^−^) identity (C3, *, *P* < 0.05, *n* = 3 *per* genotype). Countings were performed within frames sized 300 μm (h) × 100 μm (w) spanning L2-L4. Scale bar: C2, 20 μm. (PDF 135 kb) Additional file 7: Figure S7. Retention of postmitotic cells in ectopic positions in *Zbtb20* mutant cortex at E18.5. RNA ISH for TFs *Id2* (A1-A2), *Math2/NEX* (B1-B2), and *NeuroD1* (C1-D2) in DP (A1-C2) and pyriform cortex (D1-D2) of WT and mutant mice. Ectopic location of cells positive for the three TFs in the mutant is depicted by arrows. Scale bar: 200 μm. (PDF 1980 kb) Additional file 8: Figure S8. Map of the *CoupTF1* promoter and binding sites of Zbtb20. The DNA sequence of the *CoupTF1* promoter (Salas et al. [53]) contains multiple DNA binding motifs (motif_1, motif_2, motif_3) of Zbtb20 [33] . Note that Zbtb20 binds to the *CoupTF1* promoter with highest affinity at the DNA fragment ChIP_4 (as depicted in Fig. 6d1-d2), covering these motifs. (PDF 147 kb) Additional file 9: Figure S9. Expression of *Zbtb20* in *CoupTF1* ^*−/−*^ mice. ISH analysis at stage E15.5 using *Zbtb20* in situ probe on cross sections from WT (A1) and *CoupTF1* ^*−/−*^ (A2) embryo brains. Note the marked reduction of the Zbtb20 ISH signal in VP of *CoupTF1* ^*−/−*^ mutants (arrows in A2). Scale bar: 200 μm. (PDF 427 kb) Additional file 10: Table S1. Primer sequences used in the study. (PDF 35 kb)
