# Supplementary figures and images for: Zbtb20 modulates the sequential generation of neuronal layers in developing cortex
Source: Mol Brain. 2016 Jun 9;9:65. doi: 10.1186/s13041-016-0242-2 (PMC4901408; doi:10.1186/s13041-016-0242-2)

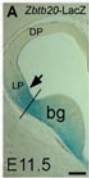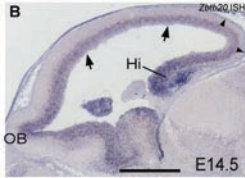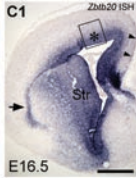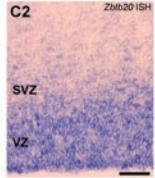

Supplement: Additional file 1: Figure S1. — Expression of TF Zbtb20 in developing pallium. (A) ß-gal staining of Zbtb20 lacZ+/− embryos at E11.5. The arrow points to a ß-gal activity expanding from the subpallium into the VZ of LP, while DP remains negative. (B) ISH on sagittal E14.5 brain sections reveals Zbtb20 expression in the entire pallial VZ (arrows). Expression in MZ is depicted by arrowheads. (C1-C2) ISH on cross E16.5 brain sections demonstrates a strong Zbtb20 expression in the pallial VZ (asterisk), gradually decreasing in the SVZ (C2). Also evident is a positive signal in the lateral migratory stream (arrow) as well as in MZ (arrowheads). The image in C2 corresponds to the boxed area in C1. BG, basal ganglia; DG, dentate gyrus, DP, dorsal pallium; Hi, hippocampus; LP, lateral pallium; Ncx, neocortex; Str, striatum; MP, medial pallium, VP, ventral pallium. Scale bars: A, 100 μm; B/C1 500 μm; C2, 100 μm. (PDF 451 kb) [file 13041_2016_242_MOESM1_ESM.pdf]

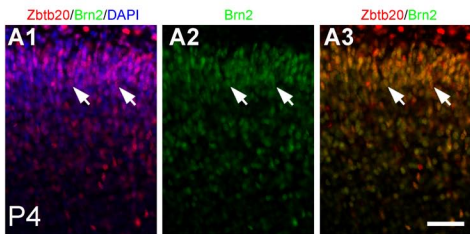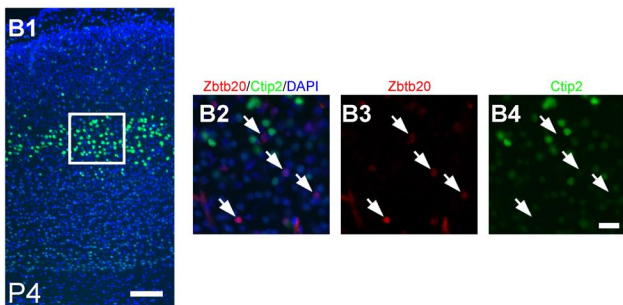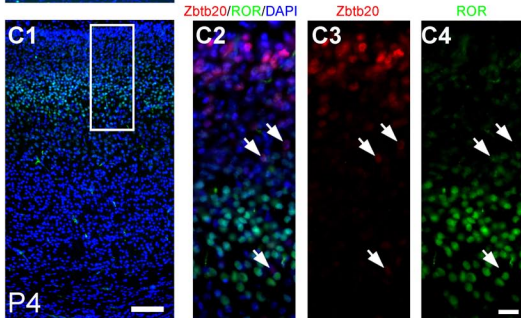

Supplement: Additional file 2: Figure S2. — Expression of TF ZBTB20 in UL neurons in early postnatal stage (P4) neocortex. (A1-A3) Double immunostaining for Zbtb20 and Brn2, and an overlay on brain cross section. Arrows depict double-positive cells at uppermost position in the developing Ncx. (B1) Immunostaining for TF Ctip2 (counterstained for DAPI) on brain cross section marks L5. The frame corresponds to the position of images shown in B2-B4. (B2-B4) Few L5 neurons express Zbtb20. Co-staining for Zbtb20 and Ctip2, and an overlay on brain cross sections. Arrows depict Zbtb20+ cells in L5 which do not exhibit co-labeling for Ctip2. (C1) Immunostaining for nuclear receptor ROR (counterstained for DAPI) on brain cross section (the strong ROR immunosignal marks L4). The frame corresponds to the position of images shown in C2-C4. (C2-C4) Co-staining for Zbtb20 and ROR, and an overlay on brain cross sections. The Zbtb20+ neurons in the upper layers do not exhibit co-labeling for ROR (arrows). Scale bars: A3, 50 μm; B1/C1, 100 μm; B4/C4, 20 μm. (PDF 161 kb) [file 13041_2016_242_MOESM2_ESM.pdf]

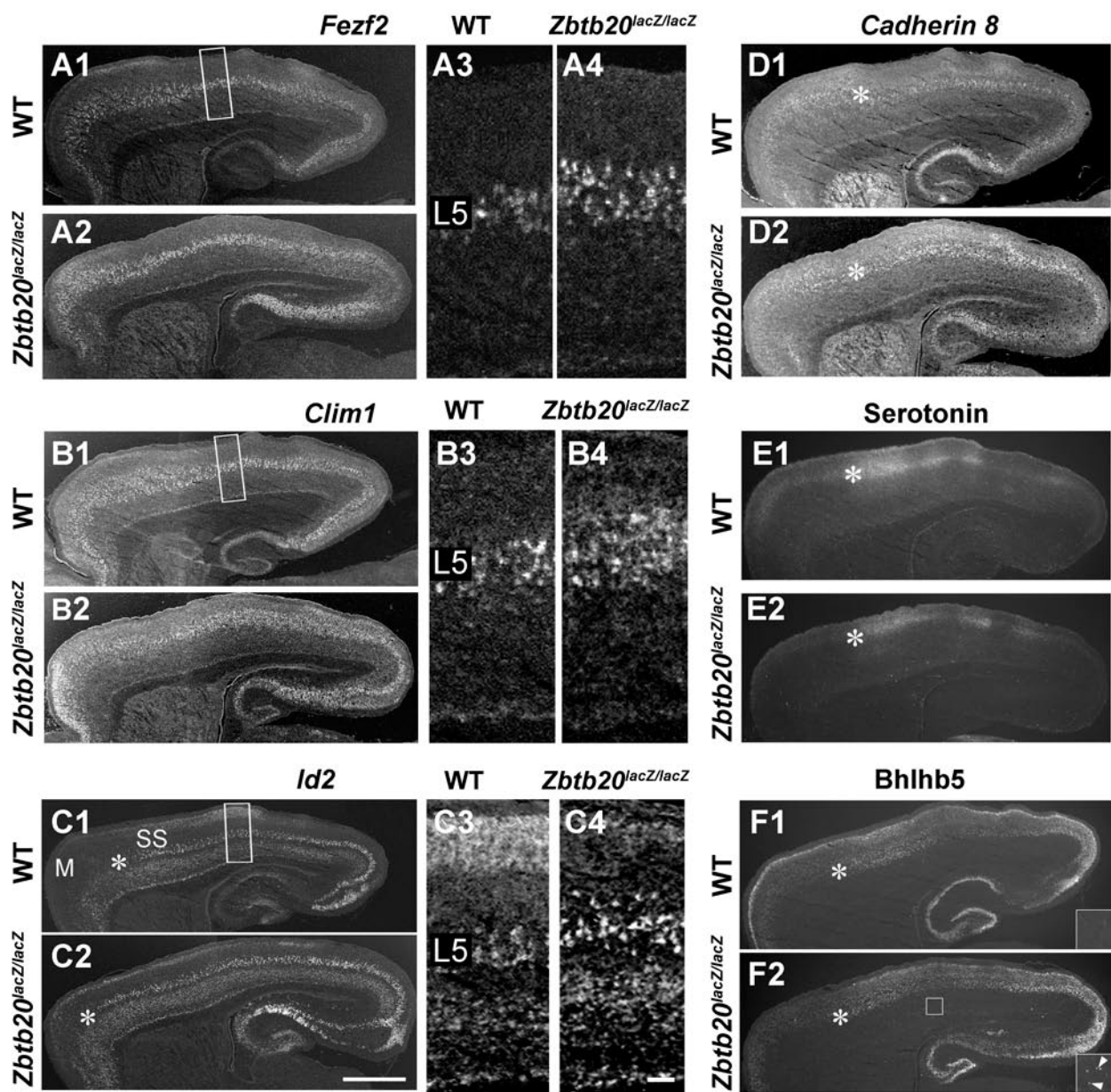

Supplement: Additional file 3: Figure S3. — Cortical arealization in Zbtb20 lacZ/lacZ mice. (A1-C4) ISH analysis on brain sagittal sections at matched levels revealing enhancement of L5 neuronal subsets, marked by Fezf2 (A1-A4), Clim1 (B1-B4) and Id2 (C1-C4), in the Zbtb20 lacZ/lacZ mutants. The pattern of Id2 expression confirms the increase of both L6 and L5 neurons, accompanied by a drastic decrease of the ULs in the Zbtb20KO cortex at P4. Asterisk in C1/C2 points to the presumptive border between the motor (M) and somatosensory (SS) area which appears rostrally displaced in the mutant. Images in A3/A4, B3/B4 and C3/C4 correspond to a field in SS cortex (indicated by a frame in A1,B1,C1). (D1-F2) However, ISH staining for Cadherin 8 (D1-D2) and IHC labelling for Serotonin (E1-E2) and Bhlhb5 (F1-F2) on sagittal sections indicates a normal position of the M/SS border (asterisk). Scale bar: C2, 1 mm. (PDF 176 kb) [file 13041_2016_242_MOESM3_ESM.pdf]

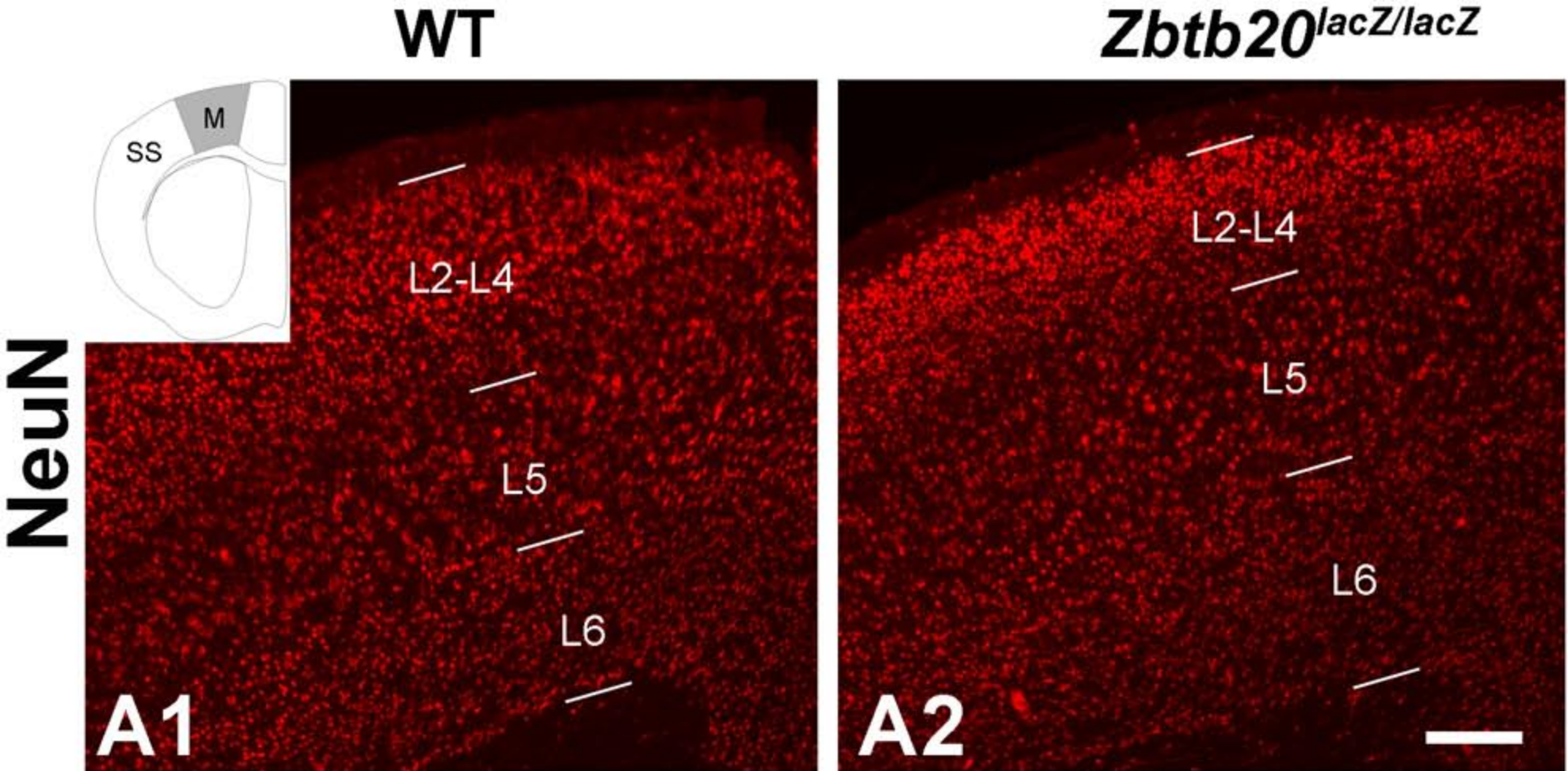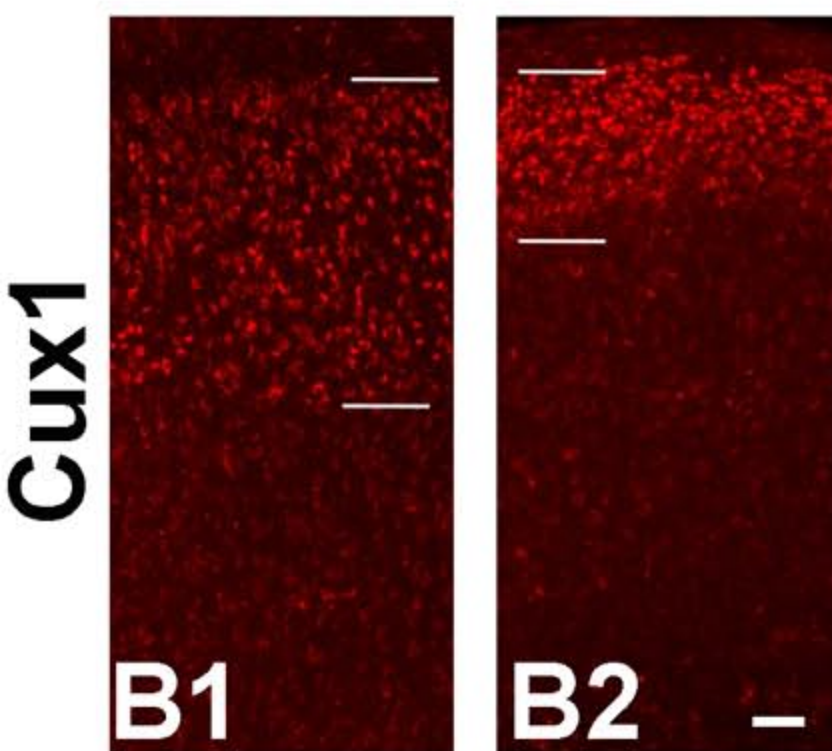

**B3** Cux1 (Motor Cortex, L2-L4)

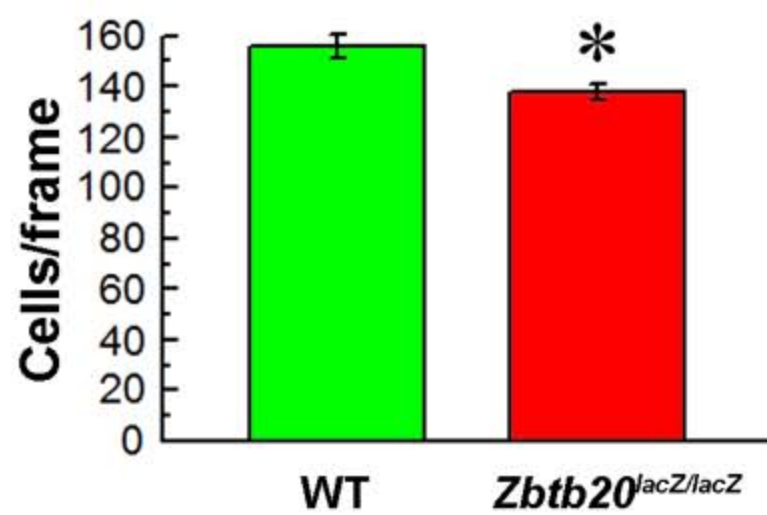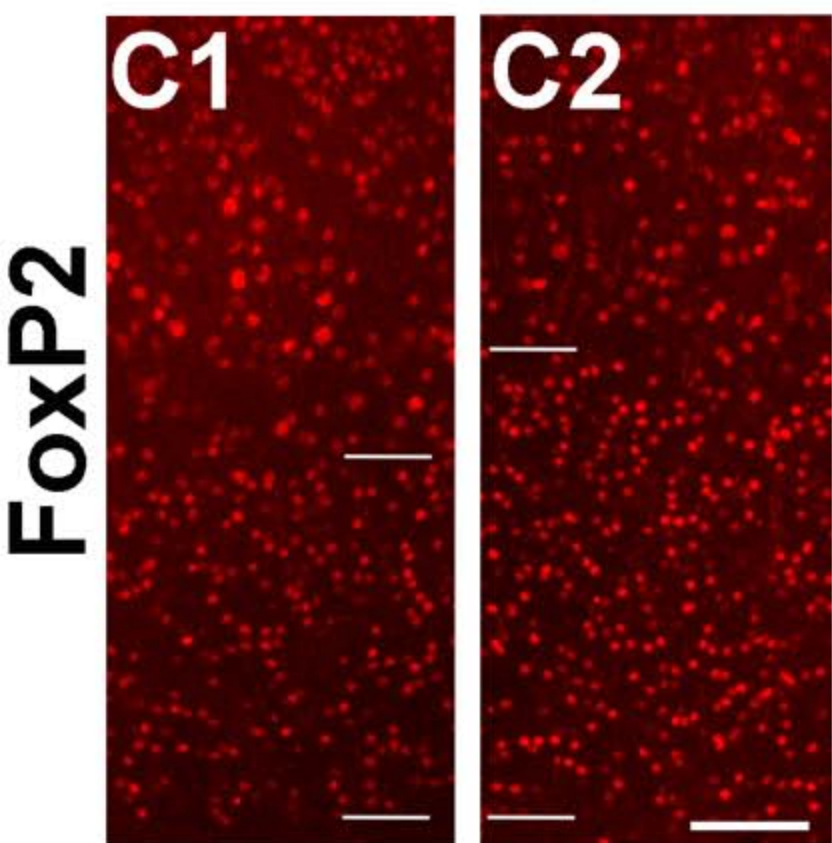

**C3** FoxP2 (Motor Cortex, L6)

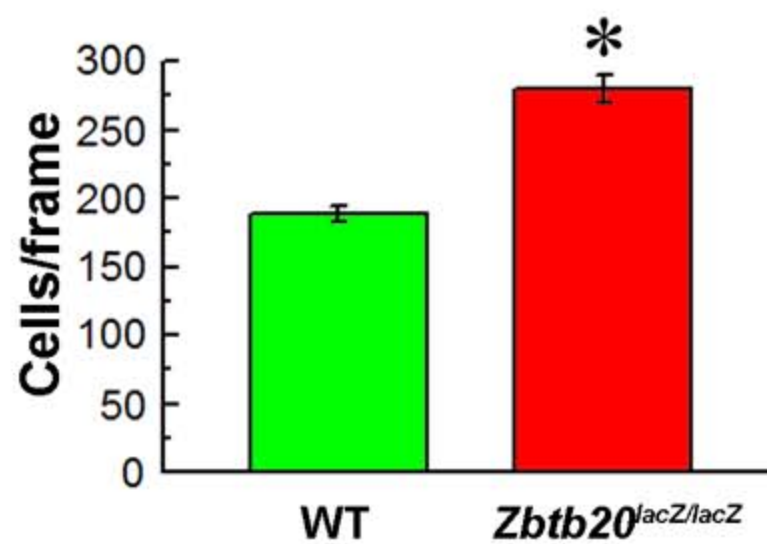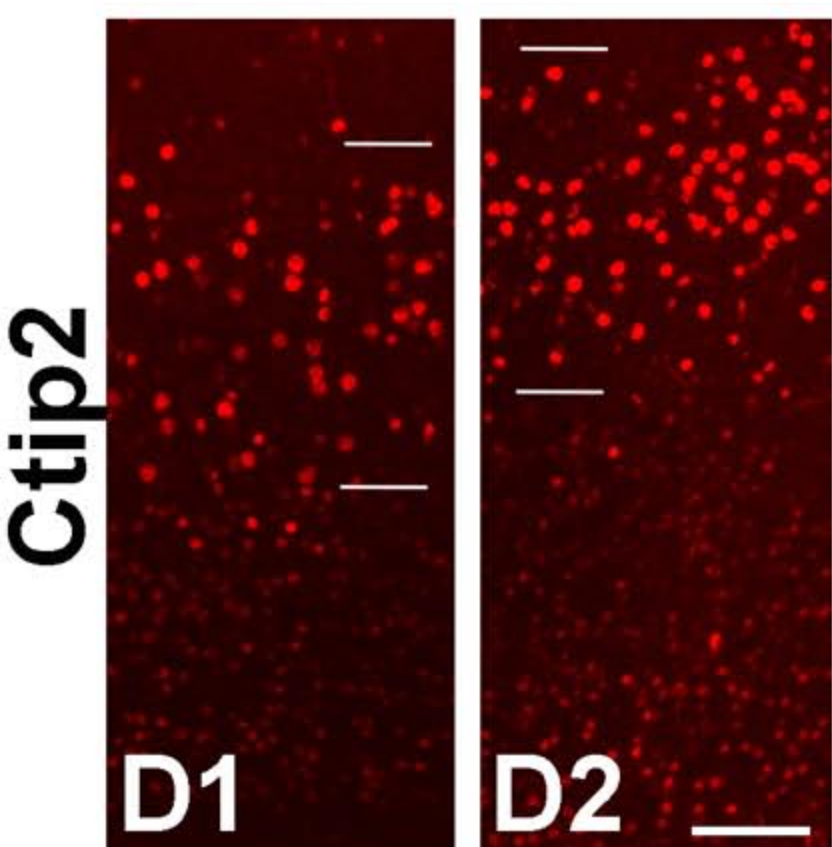

**D3** Ctip2 (Motor Cortex, L5)

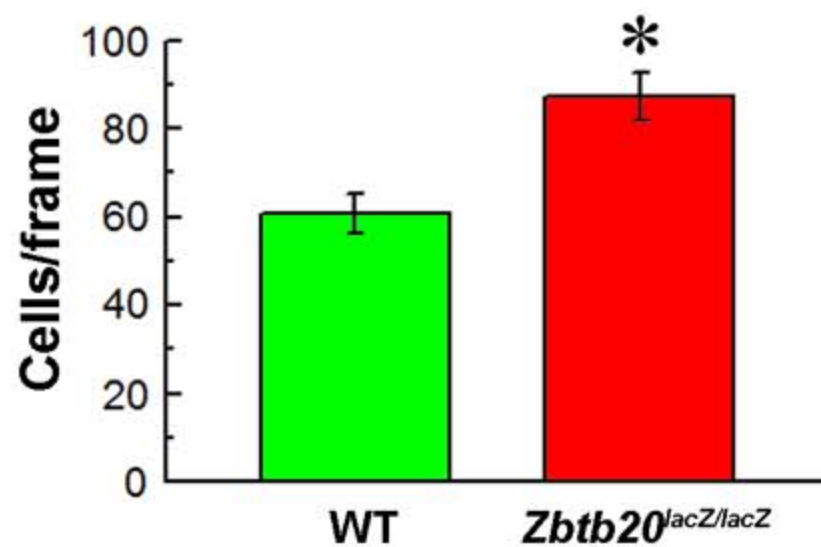

Supplement: Additional file 4: Figure S4. — Enhanced presence of lower layer neurons in primary motor cortex of Zbtb20 lacZ/lacZ mutants. (A1-A2) NeuN IHC demonstrating an overview of the motor cortex (depicted on the scheme in the upper left corner) on cross brain sections. The enlargement of L6 and L5 and the thinning of L2-L4 is apparent. (B1-B3) Decreased numbers of L2-L4 Cux1-immunostained neurons in Zbtb20-deficient cortex. (C1-D3) Immunostaining with FoxP1 (C1,C2) and Ctip2 (D1,D2) antibodies revealed in the Zbtb20 mutant an increased number of both FoxP2+ L6 and Ctip2+ L5 neurons. (B3,C3,D3) Graphs representing statistical evaluation of the results (*, P < 0.05, n = 3 per genotype). All stainings were performed at stage P8. Countings were performed within frames sized 400 μm (h) × 300 μm (w) for FoxP2, 300 μm (h) × 300 μm (w) for Ctip2, 500 μm (h) × 300 μm (w) for Cux1. Scale bars: A2, 200 μm, B2/C2/D2, 100 μm. (PDF 183 kb) [file 13041_2016_242_MOESM4_ESM.pdf]

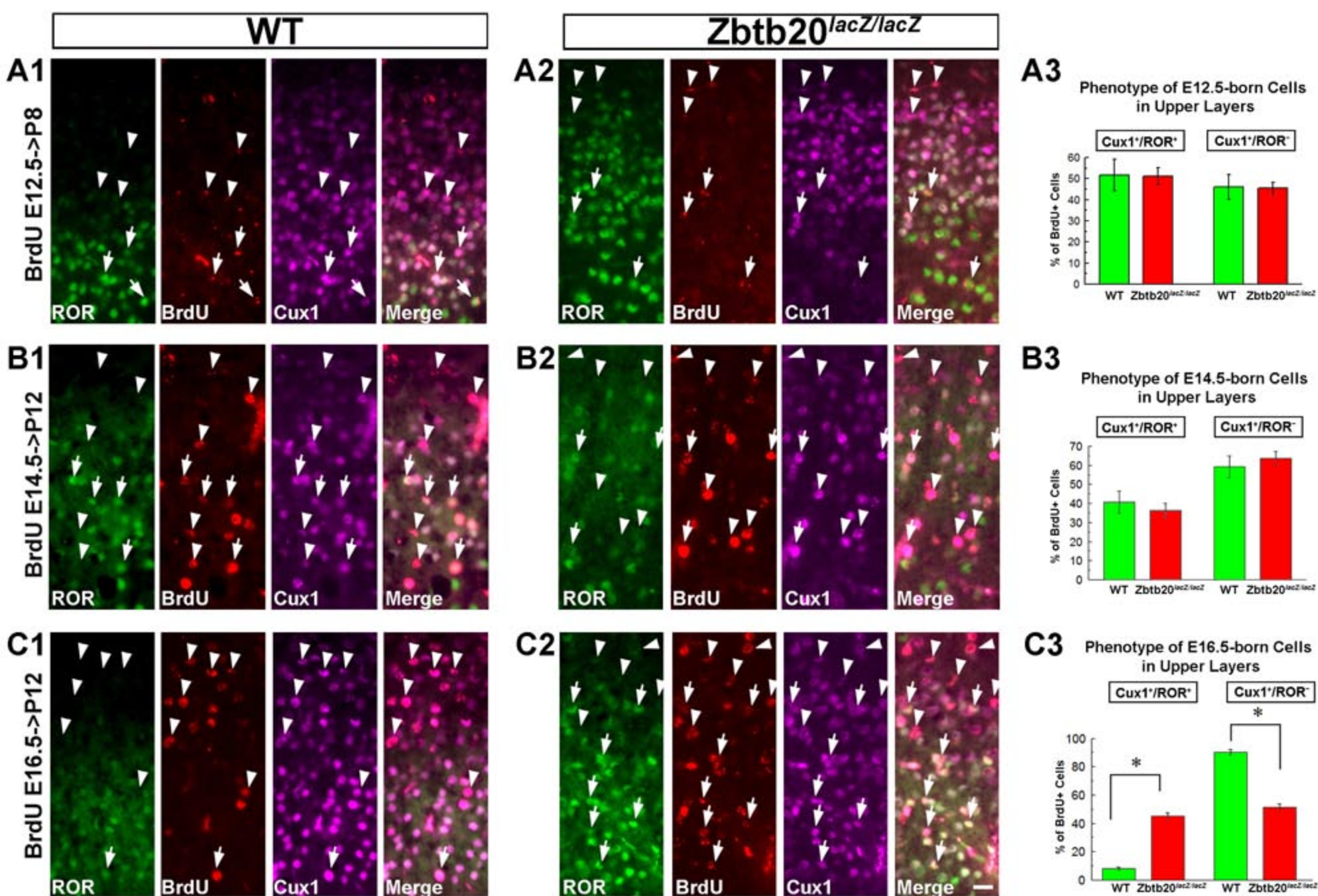

Supplement: Additional file 6: Figure S6. — Birth date analysis of specific subpopulations of neurons within the superficial (L2-L4) neocortical layers of WT and Zbtb20 lacZ/lacZ mice. BrdU was injected at E12.5, E14.5 and E16.5 and BrdU IHC was combined with Cux1 and ROR immunostaining to distinguish between L4 (Cux1+/ROR+) and L2-L3 (Cux1+/ROR−) neuronal subsets. The BrdU+/Cux1+/ROR+ and the BrdU+/Cux1+/ROR− neuronal subsets were calculated as a percentage of the BrdU+ cells within frames located in the upper layers within SS cortex. The same animals and corresponding frames were used as for Fig. 4 and Additional file 5: Figure S5. (A1-A3) Analysis of UL phenotypes born at stage E12.5. BrdU+/Cux1+/ROR+ cells are depicted by arrows, while the BrdU+/Cux1+/ROR− cells - by arrowheads. No significant differences in the proportions of Cux1+/ROR+ (L4) and Cux1+/ROR− (L2-L3) cells, born at this stage, were observed between the WT and the mutants (A3, P > 0.05, n = 3 per genotype). (B1-B3) Analysis of UL phenotypes born at stage E14.5. BrdU+/Cux1+/ROR+ cells are depicted by arrows, while the BrdU+/Cux1+/ROR− cells - by arrowheads. Similarly to stage E12.5, no significant differences in the proportions of Cux1+/ROR+ and Cux1+/ROR− cells, born at E14.5, were observed between the WT and the mutants (A3, P > 0.05, n = 3 per genotype). (C1-C3) Analysis of UL phenotypes born at stage E16.5. BrdU+/Cux1+/ROR+ cells are depicted by arrows, while the BrdU+/Cux1+/ROR− cells - by arrowheads. Note that a significantly larger proportion of Cux1+/ROR+ (L4) cells are born at stage E16.5 in the mutant ULs than in the WT ULs (C3, *, P < 0.05, n = 3 per genotype). At the same time, in the Zbtb20 deficient cortex, a significantly smaller percentage of BrdU+ cells showed L2-L3 (Cux1+/ROR−) identity (C3, *, P < 0.05, n = 3 per genotype). Countings were performed within frames sized 300 μm (h) × 100 μm (w) spanning L2-L4. Scale bar: C2, 20 μm. (PDF 135 kb) [file 13041_2016_242_MOESM6_ESM.pdf]

**WT*****Zbtb20*<sup>lacZ/lacZ</sup>***Id2***A1****A2***Math2/Nex***B1****B2***NeuroD1***C1****C2***NeuroD1***D1****D2**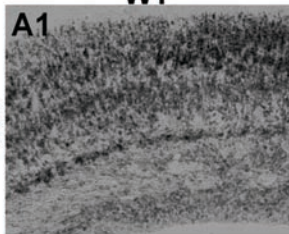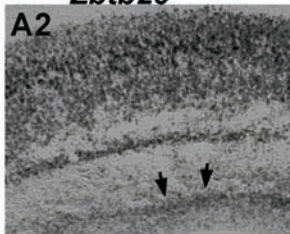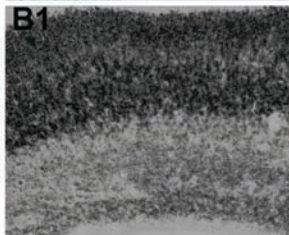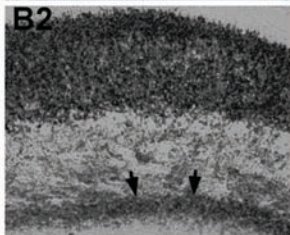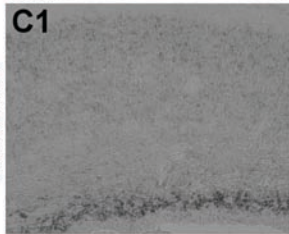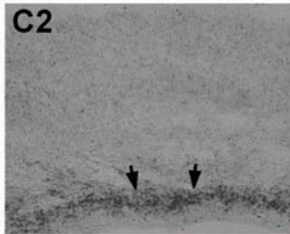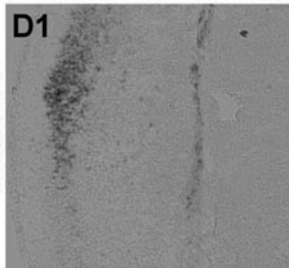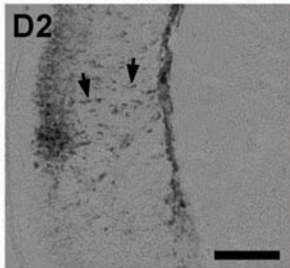

Supplement: Additional file 7: Figure S7. — Retention of postmitotic cells in ectopic positions in Zbtb20 mutant cortex at E18.5. RNA ISH for TFs Id2 (A1-A2), Math2/NEX (B1-B2), and NeuroD1 (C1-D2) in DP (A1-C2) and pyriform cortex (D1-D2) of WT and mutant mice. Ectopic location of cells positive for the three TFs in the mutant is depicted by arrows. Scale bar: 200 μm. (PDF 1980 kb) [file 13041_2016_242_MOESM7_ESM.pdf]

**WT**

***Coup-TF1*<sup>-/-</sup>**

***Zbtb20***

**A1**

**A2**

**E15.5**

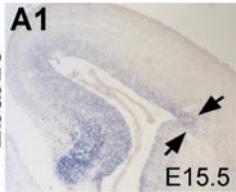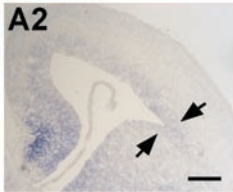

Supplement: Additional file 9: Figure S9. — Expression of Zbtb20 in CoupTF1 −/− mice. ISH analysis at stage E15.5 using Zbtb20 in situ probe on cross sections from WT (A1) and CoupTF1 −/− (A2) embryo brains. Note the marked reduction of the Zbtb20 ISH signal in VP of CoupTF1 −/− mutants (arrows in A2). Scale bar: 200 μm. (PDF 427 kb) [file 13041_2016_242_MOESM9_ESM.pdf]
