# Supplementary material for: Zbtb20 modulates the sequential generation of neuronal layers in developing cortex
Source: Mol Brain. 2016 Jun 9;9:65. doi: 10.1186/s13041-016-0242-2 (PMC4901408; doi:10.1186/s13041-016-0242-2)
Supplement: Additional file 5: Figure S5. — Distribution of neurons born at E12.5, E14.5 and E16.5 in WT and Zbtb20 lacZ/lacZ mice across neocortical layers. BrdU was injected at each of the above pointed embryonic stages and the neocortical layers were visualized by NeuN IHC. We then calculated the percentage of BrdU+/NeuN+ cells in L6, L5 and L2-L4 out of the total BrdU+/NeuN+ cells in a frame 800 μm (h) × 200 μm (w) spanning through L2-L6. The image on the left side of the figure depicts NeuN immunostaining of a WT cortex (identical with Fig. 4b,g,l) providing an overview of the cortical layers. (A1-A3) BrdU (E12.5- > P8)/NeuN double IHC demonstrates the predominant distribution of BrdU+ cells in the deep (L5-L6) layers. No significant differences between the WT and mutant mice were detected (A3, P > 0.05, n = 3 per genotype). (B1-B3) BrdU (E14.5- > P12)/NeuN double IHC depicts a larger proportion double-positive cells in deep (L5-L6) layers of the mutant cortex (arrows in B2), while a lower percentage in the superficial (L2-L4) layers (B3, *, P < 0.05, n = 3 per genotype). (C1-C3) BrdU (E16.5- > P12)/NeuN double IHC reveals a larger proportion double-positive cells in mutant deep (L5-L6) layers (arrows in C2), while a lower percentage in the superficial (L2-L4) layers (C3, *, P < 0.05, n = 3 per genotype). LL, lower neocortical layers; UL, upper neocortical layers. Scale bar: C2, 50 μm. (PDF 139 kb) [file 13041_2016_242_MOESM5_ESM.pdf]

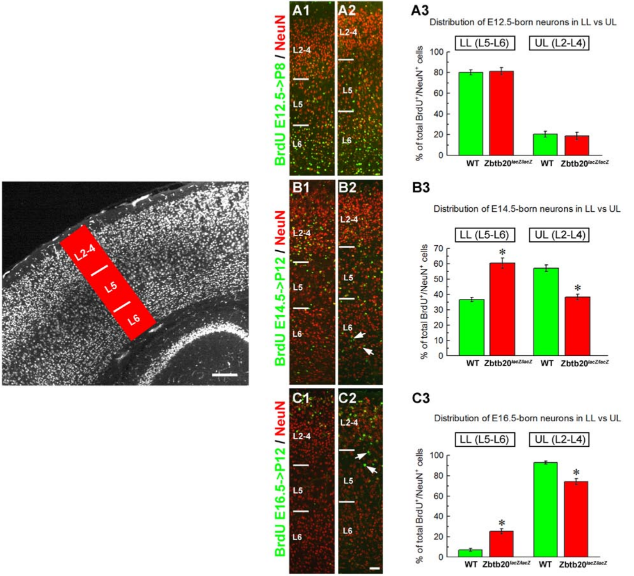

L2-4
L5
L6
WT
Zbtb20lacZ/lacZ
A1
A2
BrdU E12.5->P8 / NeuN
A3
Distribution of E12.5-born neurons in LL vs UL
LL (L5-L6)
UL (L2-L4)
% of total BrdU+/NeuN+ cells
B1
B2
BrdU E14.5->P12 / NeuN
B3
Distribution of E14.5-born neurons in LL vs UL
C1
C2
BrdU E16.5->P12 / NeuN
C3
Distribution of E16.5-born neurons in LL vs UL
