# Supplementary material for: Zbtb20 modulates the sequential generation of neuronal layers in developing cortex
Source: Mol Brain. 2016 Jun 9;9:65. doi: 10.1186/s13041-016-0242-2 (PMC4901408; doi:10.1186/s13041-016-0242-2)
Supplement: Additional file 8: Figure S8. — Map of the CoupTF1 promoter and binding sites of Zbtb20. The DNA sequence of the CoupTF1 promoter (Salas et al. [53]) contains multiple DNA binding motifs (motif_1, motif_2, motif_3) of Zbtb20 [33] . Note that Zbtb20 binds to the CoupTF1 promoter with highest affinity at the DNA fragment ChIP_4 (as depicted in Fig. 6d1-d2), covering these motifs. (PDF 147 kb) [file 13041_2016_242_MOESM8_ESM.pdf]

GTACGCGGGACCGTCCTCCTGCCTACCCCTCCTTTTGCGACCAATCACCTTCGGGAA

TGGGGTCTCAGTCACACACACCCCAACACACACACACACACACACACACACACAC

ACACCACCACCACCACCACCACCACCACCACCACCACCACCACCACCACCACCA

CCACACAGCGAGTGAGAGACTCAGTCTCTTCCTCCTCCTCCTCCTCCTCCTCTC

CCCCTCCCCCTCCCCTCCGTTTCCCACTTCTCGTCCCCTCCCCTCCTCCCCTCTCCC

TCTTCCCCGTCTTCTCGTTCGTTCGTTTGCTCTTTTCCTGTGACTGACTTGTCCGCA

CTAACAGCCGCCCCACAACAATATGAGGAGTTACAAATGCTTTATTAATAATCATTG

AAGCATTGTTTGGAGTTTGAGCATCCTGGGAATAAAAATGATGAAAAAGGAAAAAGA

GGATTGATTGGAAAGTTTATTTTAAGATCATCTTTGGGATGAATAGGAATCATCGAT

TCGGATCGAATTTGTGGCAGTAGCTGCAGTTTCATGTGTGTGCTTTGTCGTAATTAC

GCCTCCGAAACTATGATATACTTCAG**ATTTTTA**AATGAGGAGGCTTTTCATAA**TTAT**

ATAAAATGAGCGG**GATACAG**ACTAAGA**TTATAT**TGTATGAGAACTAAGATTCTAAAC

CAAGTAGAAAAAACAAATCATTAAAATG**ATG (Starting codon)**

Motif_1: ATTTTTA

Motif_2: AATA[CT]A

Motif_3: [GA]A[TC]ACAG
