# Supplementary material for: Zbtb20 modulates the sequential generation of neuronal layers in developing cortex
Source: Mol Brain. 2016 Jun 9;9:65. doi: 10.1186/s13041-016-0242-2 (PMC4901408; doi:10.1186/s13041-016-0242-2)
Supplement: Additional file 10: Table S1. — Primer sequences used in the study. (PDF 35 kb) [file 13041_2016_242_MOESM10_ESM.pdf]

Primer name	Sequence (5´ → 3´)
ChIP_1F	CTTTTGCGACCAATCACCTT
ChIP_1R	GGAGGAGGAGGAGGAAGAGA
ChIP_2F	TCTTCCTCCTCCTCCTCCTC
ChIP_2R	CGGACAAGTCAGTCACAGGA
ChIP_3F	GCCCCACAACAATATGAGGA
ChIP_3R	ACTGCAGCTACTGCCACAAA
ChIP_4F	TTCATGTGTGTGCTTTGTCG
ChIP_4R	TGGCACTGAAAACGTTAAGAAA
ChIP_5F	TCTCTCACTCGCTGTGTGGT
ChIP_5R	CTTTTGCGACCAATCACCTT
